# Organic Electrochemical Transistors for Biomarker Detections

**DOI:** 10.1002/advs.202305347

**Published:** 2024-01-23

**Authors:** Hong Liu, Jiajun Song, Zeyu Zhao, Sanqing Zhao, Zhiyuan Tian, Feng Yan

**Affiliations:** ^1^ Department of Applied Physics The Hong Kong Polytechnic University Hung Hom Kowloon Hong Kong 999077 P. R. China; ^2^ Research Institute of Intelligent Wearable Systems The Hong Kong Polytechnic University Hung Hom Kowloon Hong Kong 999077 P. R. China

**Keywords:** biomarker detections, flexible electronics, organic electrochemical transistors

## Abstract

The improvement of living standards and the advancement of medical technology have led to an increased focus on health among individuals. Detections of biomarkers are feasible approaches to obtaining information about health status, disease progression, and response to treatment of an individual. In recent years, organic electrochemical transistors (OECTs) have demonstrated high electrical performances and effectiveness in detecting various types of biomarkers. This review provides an overview of the working principles of OECTs and their performance in detecting multiple types of biomarkers, with a focus on the recent advances and representative applications of OECTs in wearable and implantable biomarker detections, and provides a perspective for the future development of OECT‐based biomarker sensors.

## Graphical abstract

This review provides a comprehensive overview of biomarker sensors based on organic electrochemical transistors (OECTs) and their associated detection methodologies. OECTs demonstrate the ability to detect a diverse range of biomarkers, exhibiting advantages including high sensitivity, biocompatibility, and flexibility. The applications of OECT‐based biomarker sensors in wearable, implantable, and portable electronics are discussed, elucidating their practical implications.

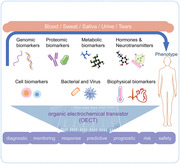

## 1 Introduction

### 1.1 Importance of Biomarker Detections

**Figure 1:**
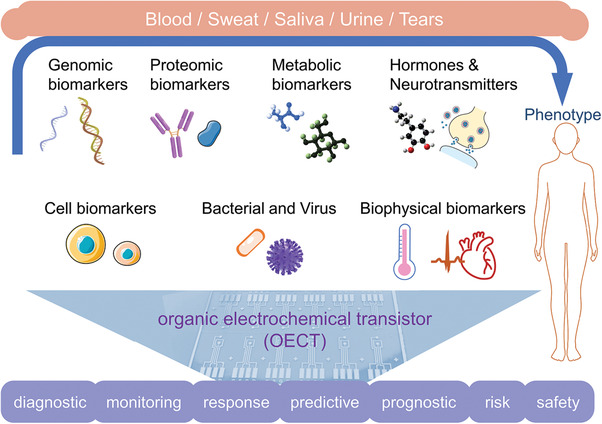
Overview of wide applications of organic electrochemical transistors in types of biomarkers detection. The graphics of synapse and cell were adapted from Servier Medical Art templates, which are licensed under a Creative Commons Attribution 3.0 Unported License; https://smart.servier.com.

Biomarkers are the indicates that can reflect whether the human body is suffering from a disease or in a normal physiological state.^[^ ^1^ ^]^ Biomarkers refer to basic physiological indicators such as body temperature, blood pressure, blood oxygen levels, and heart rate.^[^ ^2^ ^]^ They also refer to clinically relevant disease‐specific biomolecules such as nucleic acid fragments, proteins, and metabolic molecules. Biomarkers include cancer cells, pathogenic agents such as bacteria or viruses, or even electrophysiological signals that can be analyzed for the diagnosis of specific diseases (**Figure** **1**).^[^ ^3^, ^4^ ^]^ Biomarkers are classified to 7 categories (diagnostic, monitoring, response, predictive, prognostic, risk, safety) by The U.S. Food and Drug Administration and The National Institutes of Health biomarker working group according to their clinical functions,^[^ ^5^ ^]^ which are significant in indicating the potential of a disease, diagnosing a specific disease, and judging the effect of treatment. Hence, the detections of biomarkers are critical to the development of medical devices and drugs.

Genomics is positioned upstream of genetics and provides information on potential changes that may occur within an organism. The detection of genetic biomarkers facilitates the prognostication of cancer susceptibility.^[^ ^6^ ^]^ Proteins play essential roles in various biological processes, alterations in protein expression can serve as indicators of physiological or pathological changes within the body. Proteomic biomarkers can be used to diagnose diseases, including cancer, cardiovascular disease, and neurological disorders.^[^ ^7^ ^]^ For example, carcinoembryonic antigen (CEA) is a protein biomarker that is used to monitor the progression of types of cancer, including colon, lung, and breast cancer. Metabolites, such as blood glucose, can serve as biomarkers to diagnose and monitor obesity and diabetes. Furthermore, some small molecules such as hormones and neurotransmitters can serve as biomarkers for metabolic abnormalities and mental health disorders.^[^ ^8^ ^]^ In addition to molecular biomarkers, changes in cellular morphology and the presence of antigens such as bacteria and viruses can be utilized for disease diagnosis. One notable example is the detection of the coronavirus, which is used to diagnose virus infections.^[^ ^9^ ^]^ Electrophysiology biomarkers are biomarkers that are based on the electrical activity of cells and tissues in the body. Electrophysiological biomarkers can be used to diagnose and monitor various conditions, including neurological disorders, cardiovascular disease, and muscular disorders. For example, electroencephalography (EEG) can be used to detect abnormal brain activity in conditions such as epilepsy and sleep disorders, while electrocardiography (ECG) can be used to detect abnormalities in heart function.^[^ ^10^, ^11^ ^]^

### 1.2 Biomarker Detection Methods

Various types of biomarkers necessitate different detection methods. Nucleic acid detection methods mainly include polymerase chain reaction (PCR) technology, reverse transcription PCR (RT‐PCR), and fluorescence in situ hybridization (FISH), while protein detection methods mainly include enzyme‐linked immunosorbent assay (ELISA), immunohistochemistry (IHC), and mass spectrometry.^[^ ^12^, ^13^, ^14^ ^]^ Cell detection methods primarily involve microscopy, although molecular detection methods such as PCR or IHC can also be used to identify cell types due to the diverse composition of cells. Bacteria and virus detection often rely on testing their nucleic acids or antigenic proteins, thus methods such as PCR, ELISA, and immunofluorescence assay are commonly employed.^[^ ^15^, ^16^ ^]^ Culture‐based methods, which involve the growth of viruses or bacteria in a laboratory culture medium, are also used for detecting bacteria and viruses. The detection of electrophysiological signals is based on the measurement of electrical activity generated by biological systems. Electrical signals are usually derived from the body surface or inside the body with wires, and then amplified and recorded with instruments. In addition, there are electrochemical and optical biosensors that serve as sensing tools, capable of detecting a diverse range of biological targets such as DNA/RNA, proteins, cells, viruses, and bacteria. Electrochemical sensing methods comprise amperometric, potentiometric, impedimetric, and voltammetric biosensors, while optical biosensors include surface plasmon resonance biosensors, fluorescence biosensors, and luminescence biosensors.

PCR is a widely used method for amplifying a specific DNA or RNA sequence. It involves a series of temperature cycles to denature and anneal the nucleic acid template, and the use of a thermostable DNA polymerase enzyme to synthesize new strands of DNA or RNA.^[^ ^17^ ^]^ RT‐PCR is a variant of PCR that enables the amplification of RNA sequences.^[^ ^18^ ^]^ It involves the use of a reverse transcriptase enzyme to convert RNA into complementary DNA (cDNA), which can then be amplified by PCR. PCR technology has high sensitivity and specificity, and can detect a single copy of the target sequence, making it the gold standard for nucleic acid detection. Moreover, PCR technology is generally applicable to all organisms with nucleic acid, and can diagnose diseases or predict potential disease threats by detecting the sequence of nucleic acid biomarkers. However, PCR is prone to contamination, and even minute amounts can lead to false‐positive results, necessitating precautions to avoid contamination. Additionally, PCR can be expensive, especially if multiple reactions are required, and the equipment and reagents involved can be costly. Finally, PCR requires trained professionals to perform it in a specialized laboratory.

ELISA is the most commonly used method for detecting and quantifying specific protein biomarkers.^[^ ^19^, ^20^ ^]^ It involves the use of a capture antibody that binds to the target protein, followed by the addition of a detection antibody that is conjugated to an enzyme. The enzyme catalyzes a colorimetric or chemiluminescent reaction that can be measured using a spectrophotometer or a luminometer. ELISA is the gold standard for protein biomarker detection, providing high sensitivity and the ability to quantitatively detect small amounts of specific antibodies or antigens in a sample. However, ELISA labeling steps can be cumbersome and time‐consuming, and the technique has a limited dynamic range, which means that very high or very low concentrations of antigens or antibodies may not be accurately detected.

IHC is a widely used technique in pathology and research for visualizing and localizing specific proteins or antigens in tissue samples.^[^ ^21^ ^]^ IHC involves the use of specific antibodies that bind to the target protein or antigen, followed by the detection of the antibody using a visual marker, such as a fluorescent dye or chromogen, which produces a visible signal. IHC can visualize and localize specific proteins or antigens within a tissue sample, providing information on their distribution and cellular localization.

Biosensors employ diverse mechanisms to enable the qualitative and quantitative analysis of analytes. To facilitate detection, specific recognition receptors, such as antibodies, enzymes, or aptamers, are typically immobilized on a substrate surface.^[^ ^22^, ^23^ ^]^ Electrochemical biosensors can detect a wide range of biological molecules, including glucose, cholesterol, nucleic acid, and proteins.^[^ ^24^ ^]^ Amperometric biosensors measure the changes in current resulting from the reaction between the analyte and the immobilized recognition receptors. Potentiometric biosensors measure the changes in voltage, while impedimetric biosensors measure the changes in impedance. Optical detection relies on the exploitation of the interaction between the optical field and a biorecognition receptor to facilitate the analysis and measurement of analytes.^[^ ^25^ ^]^ However, the portability and wearability of optical biosensors may be difficult due to their dependence on specific wavelength range light sources and specialized detection equipment.

It is challenging to have a comprehensive approach that can accurately detect a broad range of biomarkers, and organic electrochemical transistors (OECTs) offer a promising solution.^[^ ^26^, ^27^, ^28^, ^29^, ^30^ ^]^ OECTs are capable of converting biological signals into electrical signals and amplifying them in situ, making them highly sensitive for detection.^[^ ^31^, ^32^ ^]^ Furthermore, OECTs exhibit excellent stability in liquid detection and can be operated at low voltages.^[^ ^33^, ^34^ ^]^ Their flexibility and biocompatibility make them well‐suited for use in wearable and implantable electronics. OECTs have broad applications in the detection of various types of biomarkers, including nucleic acids, proteins, metabolites, neurotransmitters, hormones, cells, bacteria, viruses, and electrophysiological signals.^[^ ^28^, ^30^ ^]^

## 2 Work Mechanism and Features of OECTs

### 2.1 OECT Device Structure and Mechanism

**Figure 2:**
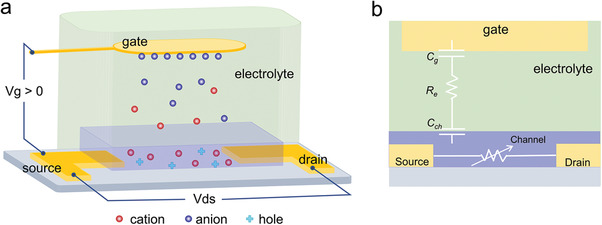
a) Device structure of a typical OECT with a polarizable gate electrode. b) The electronic circuit and ionic circuit of OECTs.

An OECT is composed of three electrodes, including a gate, source, and drain, an organic semiconductor channel, and an electrolyte that connects the gate and channel. In the case of a typical depletion‐mode OECT, as shown in **Figure** **2**a, a positive gate voltage is applied, causing cations in the electrolyte to migrate into the hole‐rich channel, switching the channel from an on‐state to an off‐state. For field‐effect transistors, which rely on the electric field of the dielectric layer to modulate carrier concentration, the operation voltages are usually tens of volts. However, OECTs can be operated using very low voltages (less than 1 volt). In OECT, the ion permeable channel enables high volume capacitance. Therefore, a small gate voltage can drive a large channel current, facilitating efficient gate modulation.

The device performances of OECTs are primarily determined by the device's geometric dimensions and the figures of merit of the channel materials. Figure 2b illustrates the electronic and ionic circuits in OECTs, which help to explain the relationship between the device performance and its underlying physics. In the electronic circuit, the channel is considered a resistor that is modulated by gate voltage. In the ionic circuit, the gate capacitance *C_g_*, electrolyte resistor *R_e_*, and channel capacitance *C_ch_* are connected in series to form an RC circuit. For effective gating, the gate capacitance *C_g_* should be significantly larger than the channel capacitance *C_ch_*, which ensures that most of the gate potential is applied to the channel. With the exception of nonpolarizable gate electrodes (e.g., Ag/AgCl), larger gate area or thicker poly(3,4‐ethylenedioxythiophene) polystyrene sulfonate (PEDOT:PSS) films can be used to achieve a high gate capacitance.^[^ ^35^ ^]^ The state of the electrolyte (e.g., liquid, gel, or solid), ion concentration, and ionic radius could affect the migration of ions in the solution and conjugated polymer and thereby have impact on the device performance.^[^ ^36^ ^]^

### 2.2 Features of OECTs

#### 2.2.1 Simple Device Structure

The structure of a basic OECT is relatively simple compared to field effect transistors (FETs) and bipolar junction transistors. Instead of using an insulating layer, the OECT uses the electrolyte layer that can be filled with bodily fluids such as urine and sweat, as well as solutions such as NaCl and phosphate buffered saline (PBS). In an OECT biosensor, the gate electrode can be integrated with the channel on the same substrate or separated from it. Regardless of the gating configuration, biomolecules can be conveniently modified on bio‐recognition sites (such as gate surface, channel surface, or in electrolyte).^[^ ^37^, ^38^, ^39^, ^40^, ^41^ ^]^ Separating the gate as a bio‐recognition site is convenient for bio‐modifications and protects the channel from being contaminated.

Furthermore, organic materials can be deposited using simple solution‐phase techniques such as spin‐coating or ink‐jet printing, whereas FETs require more complex fabrication processes such as deposition and etching. This reduces the cost of instruments and materials and facilitates large‐scale preparation and industrialization. Additionally, the simple device structure and few fabrication steps of OECTs make them easier to incorporate into fiber‐based and flexible devices, while maintaining high electrical performance.^[^ ^42^, ^43^ ^]^

#### 2.2.2 Low Working Voltages and High Transconductance

The high volumetric capacitance of the channel enables the low working voltages of OECTs, which can prevent the risk of water electrolysis in aqueous samples. Additionally, the low voltage could preserve the structural integrity of biological molecules, including proteins, while minimizing the potential harm to cell analytes during testing. Low applied voltages in OECTs not only reduce the risk of harm to the human body during testing but also make OECTs attractive for low power consumption electronic devices.

The transconductance of an OECT biosensor is a critical parameter that reflects its ability to efficiently amplify electrical signals. A higher transconductance can lead to greater amplification of weak signals, resulting in improved signal‐to‐noise ratios and higher detection sensitivity. This characteristic is particularly important in biosensing applications, especially for detecting biomarkers that are present at very low concentrations in the body. The transconductance of OECTs exceeded those of state‐of‐the‐art metal oxide semiconductor FETs (MOSFETs) and FETs based on two‐dimensional materials.^[^ ^31^, ^44^, ^45^, ^46^ ^]^ It is also higher than that of solution‐gated FETs because of the high volumetric capacitance. The transconductance of OECT at the saturation region is expressed as: ^[^ ^35^ ^]^

(1) $$g_{m}=\frac{W}{L}\;\mu dC^{*}\left(V_{\mathrm{TH}}-V_{\mathrm{GS}}\right)$$

where W is channel width, L is channel length, d is channel thickness, µ is the electronic carrier mobility, and C* is the capacitance of the channel per unit volume, *V_th_* is the threshold voltage. Except for the channel geometric dimensions *Wd/L*, the transconductance of OECT is positive correlated to µC* value, which is a materials‐system‐dependent product.

Regarding channel materials, PEDOT:PSS is a highly attractive material, which exist both high µC* value of ≈84 F cm^−1^ V^−1^ s^−1^ and high stability.^[^ ^47^ ^]^ Through crystallization,^[^ ^33^ ^]^ using higher molecular weight of PSS,^[^ ^48^ ^]^ and incorporating additives,^[^ ^49^, ^50^ ^]^ the µC* value of PEDOT:PSS can be increased to nearly 500 F cm^−1^ V^−1^ s^−1^. Other p‐type organic semiconductors, such as poly(2‐(3,3′‐bis(2‐(2‐(2‐methoxyethoxy)ethoxy)ethoxy) [2,2′‐bithiophen]−5‐yl)thieno[3,2‐b]thiophene) (p(g2T‐TT)),^[^ ^36^, ^51^, ^52^ ^]^ poly(2‐(4,4′‐bis(2‐methoxyethoxy)−5‐methyl‐[2,2′‐bithiophen]−5‐yl)−5‐methylthieno[3,2‐b]thiophene) (pgBTTT),^[^ ^53^ ^]^ p(gT2) homopolymer derived p(g2T2‐g4T2),^[^ ^54^ ^]^ and diketopyrrolopyrrole (DPP) derivative PTDPP–DT,^[^ ^55^ ^]^ have demonstrated comparable high µC* values. In addition, high transconductance can be achieved by designing the channel size, such as increasing the thickness of the polymer film and the channel width.^[^ ^50^, ^56^, ^57^ ^]^ The low carrier mobility of n‐type organic semiconductors makes the performances of n‐type OECT lag behind. Nevertheless, recent advancements in materials development have led to the discovery of new materials with improved n‐type carrier mobility. ^[^ ^58^, ^59^, ^60^, ^61^, ^62^ ^]^

#### 2.2.3 Biocompatibility

For cell detection and in vivo implant testing, it is important to consider the biocompatibility of the devices. OECT devices are primarily composed of thin (tens of nanometers) gold or platinum films serving as electrodes, organic semiconductor films, and substrates. Gold and platinum are medical‐grade precious metals that possess unique biocompatibility characteristics, good ductility, and are non‐toxic to the human body.

Conductive polymers have great potential for use in a wide range of biocompatible applications such as tissue engineering, drug delivery, and biosensors because they can interact with biological systems, stimulate cell growth, and promote tissue regeneration without causing significant harm.^[^ ^63^, ^64^ ^]^ The chemical structure of some conducting polymers is similar to natural tissue components such as proteins, which makes them less likely to trigger immune reactions.^[^ ^65^ ^]^ In OECT biosensors, conductive polymers show unique physicochemical properties, structural tunability, mechanical compatibility, and the ability to support cellular activities.^[^ ^41^, ^66^, ^67^ ^]^ PEDOT:PSS has been demonstrated to possess favorable biocompatibility characteristics in numerous cell detection and electrophysiological testing studies.^[^ ^41^, ^68^, ^69^, ^70^ ^]^ Various surface treatments and customization techniques can be employed to enhance cell adhesion and facilitate the anchoring of viruses and chemicals to conductive polymers.^[^ ^39^, ^68^, ^71^ ^]^

The biocompatibility of kinds of substrates used in OECT devices for in vivo detection has also been demonstrated. Ultra‐thin parylene film with embedded OECTs caused no noticeable glial scar after a one month implantation in the rat neocortex.^[^ ^72^ ^]^ OECTs on ultra‐thin parylene substrate were attached on rat cortical surface to test electrocorticography (ECoG) signals.^[^ ^70^ ^]^ The use of a honeycomb grid parylene substrate with a low Young's modulus and high stretchability enhances the biocompatibility of OECT devices. Furthermore, the application of an antithrombotic coating of poly(3‐methoxypropyl acrylate) on the outermost layer improves the blood compatibility of the devices, resulting in higher signal noise ratio and longer monitoring time of in vivo ECG.^[^ ^73^ ^]^ To minimize tissue damage, a biodegradable OECT array was developed using biocompatible materials such as poly(lactic‐co‐glycolic acid)), Au, PEDOT:PSS, and a water‐soluble polyvinyl alcohol (PVA) film. The OECT array exhibited the ability to detect low‐amplitude electrocorticography (ECoG) signals in rat brain under various physiological conditions, while preserving tissue integrity for over 48 hours.^[^ ^74^ ^]^

#### 2.2.4 Flexibility

The flexibility of a device is determined by several factors, including the device structure, substrate materials, semiconductor materials, and others. The challenge is to fabricate devices on flexible substrates while maintaining stable device performance. This involves the prevention of fractures in electrodes or semiconductors on flexible substrates and the maintenance of conductivity even in the presence of cracks in devices. Inorganic semiconductors typically exhibit a crystalline structure that imparts brittleness and limited flexibility. In contrast, organic semiconductors used in OECT devices have covalent bonds that enable the organic molecules to rotate and bend, resulting in a high degree of flexibility.^[^ ^75^ ^]^ In addition, compared to traditional silicon‐based transistors, organic transistors are easier to be fabricated on flexible substrates such as plastics, paper, and textiles, through low‐cost fabrication process, which uses solution‐based techniques such as printing or coating.

In addition to the contribution of organic semiconductors, the use of ion gels and solid electrolytes promotes the flexible applications of OECTs. Ion gels are soft materials composed of a polymer matrix and an ionic liquid. The polymer matrix provides mechanical support and stability, whereas the ionic liquid enables ion transport.^[^ ^76^, ^77^, ^78^, ^79^, ^80^, ^81^ ^]^ Solid polymer electrolytes are a class of materials that are composed of a polymer matrix and an inorganic salt, and are used to conduct ions through the polymer chain. Commonly used polymers for fabrication of solid‐state OECT include polyvinylidene fluoride, polyvinylidene fluoride‐hexafluoropropylene copolymer, polyvinyl alcohol, and biocompatible polymers such as chitosan and cellulose.^[^ ^82^, ^83^, ^84^, ^85^ ^]^

Flexible transistors are used in various bio applications with the formation of ultra‐thin patches and fibers.^[^ ^42^, ^74^, ^86^, ^87^ ^]^ The flexible OECT biosensor is a promising technology for use in wearable or implantable medical devices, which can be combined with other electronic components to form an integrated bioelectronic system for real‐time monitoring and analysis of biomarkers.

## 3 Biomarker Detections Based on OECTs

Biomarker detection based on OECTs is a rapidly developing field with promising applications in various areas of healthcare. The detection of biomarkers in bodily fluids, including blood, urine, and saliva, offers valuable diagnostic information. However, many biomarkers are present at very low concentrations, making their detection challenging. OECTs can interface with biological systems, enabling the detection of biomolecules such as proteins, nucleic acids, and small molecules with high sensitivity and low detection limits. These properties make OECTs promising for early disease diagnosis. Most biomarker detection based on OECTs are label‐free, which simplifies the detection process and minimizes the risk of being interfered with labels. Biomarkers can be categorized into four major groups based on the type of analytes, including molecules, cells, bacteria and viruses, and biophysical characteristics.

**Figure 3:**
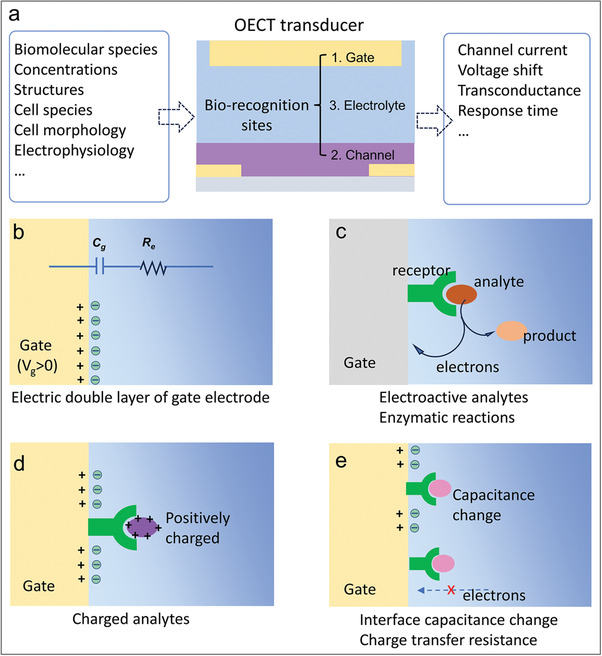
Mechanisms of OECT biosensor. a) OECT as a transducer converts bio signals into electrical signals. b) Electric double layer of the gate electrode of an OECT. c) Sensing of electroactive analytes and enzymatic reactions. d) Sensing of charged analytes. e) Analytes change the interface capacitance and the charge transfer resistance.

OECTs are capable of converting the biological signals of analytes into electrical signals, typically observed as variations in channel current and shifts in transfer curves. (**Figure** **3**a). The principle of OECT biosensors varies depending on the type of analyte and the experimental design. In Figure 3b–e, the gate electrode serves as an example of the recognition site in biosensing. For electroactive analytes and enzymatic reactions, electrons of redox reactions transfer to gate electrode, leading to changes in the gate potential.^[^ ^88^ ^]^ The detection of metabolites, such as glucose, is a prevalent application of redox reaction‐based OECT biosensors.^[^ ^89^ ^]^ Furthermore, enzymes can be employed as labels to introduce redox reactions and amplify signals, capitalizing on the significant signal enhancement resulting from charge transfer.^[^ ^37^ ^]^ In addition to redox reactions, charged analytes can also induce changes in the gate electrode potential through electrostatic interactions.^[^ ^90^ ^]^ Moreover, when analytes are modified on the gate electrode, they can alter the interface capacitance and charge transfer resistances, subsequently altering the effective gate voltage. These variations in the gate voltage can be detected and quantified, enabling the sensitive detection and analysis of various biomolecular interactions. The principle of using the channel as a recognition site is similar to that of the gate electrode, but the mechanism of biosensing involving reactions at the channel is more complex. ^[^ ^91^ ^]^

### 3.1 Molecular Biomarker Detections

Molecular biomarkers are analytes composed of a single component and can be classified into several categories based on their biological makeup, including genomic biomarkers, proteomic biomarkers, metabolic biomarkers, hormones, and neurotransmitters. Proteins are typically macromolecules, while metabolites, hormones, and neurotransmitters are usually small molecules. The surface of an OECT can be modified with specific biological recognition receptors, such as antibodies, enzymes, or aptamers, to anchor the analyte being detected. The binding of the biomolecules to the receptor on the OECT surface induces a change in the device's electrical properties, which can be detected, measured, and quantified.

#### 3.1.1 Genomic Biomarkers

Genomic biomarkers are variations in DNA or RNA that can be measured as indicators of disease or response to treatment. These biomarkers are generally identified through genetic analysis techniques, such as polymerase chain reaction (PCR).^[^ ^17^ ^]^ By offering a precise and direct approach for disease diagnosis and prediction, genomic biomarkers have become a valuable tool in clinical practice. Genomics is situated upstream of genetics and has the potential to reveal the possible alterations that may occur in an organism.

**Figure 4:**
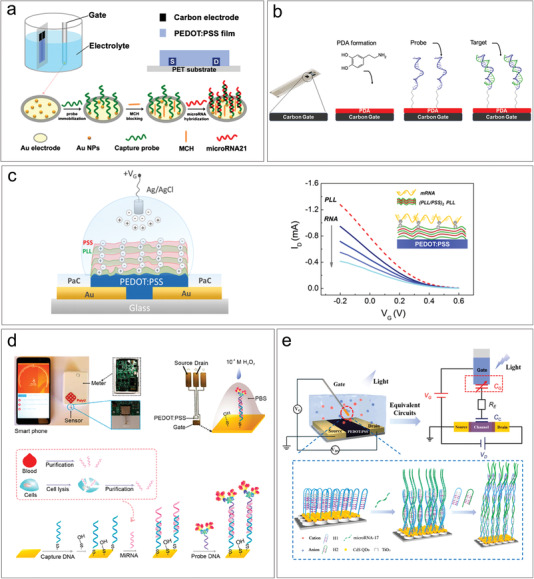
Genomic biomarkers detection based on OECTs. a) The detection of microRNA21 through hybridization with the capture DNA modified on Au NPs. Reproduced with permission.^[^ ^92^ ^]^ Copyright 2018, Springer Nature. b) The detection of an oligonucleotide through covalent binding between the quinone groups on the gate electrode. Reproduced with permission.^[^ ^93^ ^]^ Copyright 2022, Wiley‐VCH. c) The detection of mRNA by electrostatic adsorption. Reproduced with permission.^[^ ^94^ ^]^ Copyright 2017, American Chemical Society. d) The detection of microRNA 21 through labeling of a nanoprobe composed of Au NPs and HRP. Reproduced with permission.^[^ ^95^ ^]^ Copyright 2021, American Chemical Society. e) The detection of miRNA‐17 using an OPECT platform and nucleic acid amplification techniques. Reproduced with permission.^[^ ^97^ ^]^ Copyright 2022, Elsevier.

The principle of nucleic acid biosensors generally involves immobilizing a single‐stranded probe DNA at the at the sensor interface to complement with the target DNA/RNA to be detected. The fixing of the probe DNA can be conducted through modifying the sensor interface with functional groups by salinization or gold‐thiol contacts. In the study of Peng et al., Au nanoparticles were electrodeposited onto gold gate electrodes to immobilize a 5′‐thiol group‐terminated DNA capture probe (**Figure** **4**a).^[^ ^92^ ^]^ This sensor was developed for the purpose of detecting microRNA21, which is a common gene biomarker associated with various types of cancer and heart disease. An all‐printed OECT sensor was developed by Sensi et al. for the detention of an oligonucleotide containing the HSP70 promoter CCAAT sequence, which is known to be associated with cancer progression and aggressiveness when overexpressed (Figure 4b).^[^ ^93^ ^]^ The immobilization of the DNA probe was achieved through covalent binding between the quinone groups of a polydopamine film on the gate electrode and the 5′‐amino terminal group of the probe DNA. Because RNA is negatively charged, it can also be modified on channel through electrostatic adsorption with positively charged films (Figure 4c).^[^ ^94^ ^]^

Through labeling, ultrasensitive analysis of microRNA 21 was demonstrated in Fu et al.’s work.^[^ ^95^ ^]^ As shown in Figure 4d, SH‐modified capture DNA was immobilized on Au gate electrode via a strong SH‐Au binding. Then, target microRNA21 was added to react specifically with the capture DNA. To achieve higher detection sensitivity, microRNA 21 was further reacted with a probe DNA labeled with a nanoprobe composed of Au nanoparticles and horseradish peroxidase (HRP). Upon the addition of H_2_O_2_, direct electron transfer occurs between the nanoprobe and the electrode surface. The amount of electron transfer and gate potential variation is ultimately dependent on the concentration of microRNA 21. This platform shows good selectivity and a low detection limit down to 10 fM. The ultrasensitive sensor was further used to detect miRNA expression in breast cancer cells and normal cells, and the results showed that microRNA 21 concentrations in breast cancer cells were higher than those in normal cells. Due to the sensitivity of OECTs to charge transfer, labeling with hydrogen peroxide can be used to introduce a redox reaction, thereby increasing the detection sensitivity.

Organic photoelectrochemical transistors (OPECTs) are a new sensing technology that has been developed in recent years based on OECTs.^[^ ^96^ ^]^ Gao et al. demonstrated the detection of miRNA using an OPECT detection platform.^[^ ^97^, ^98^ ^]^ MiRNA‐17 is an important biomarker associated with various types of cancer, including lung cancer, breast cancer, and gastric cancer. In this platform, the target miRNA‐17 could open the hairpin DNA (H1) modified on the gate electrode, and trigger the reaction with H1 and H2 and eventually forming a long double stranded helix with repeating units of H1 and H2 (Figure 4e). This sensing strategy utilized nucleic acid amplification techniques to amplify the originally weak biological signal, ultimately achieving highly sensitive detection of miRNA‐17 with a detection limit of 1 pM. This detection method can be applied to other types of nucleic acid detection due to its versatility.

#### 3.1.2 Proteomic Biomarkers

Proteins are often preferred as biomarkers for several reasons:^[^ ^99^, ^100^ ^]^ 1) Proteins are abundant in biological samples, and their expression levels can vary significantly in response to different physiological or pathological conditions. 2) Proteins are structurally diverse and can perform a range of functions in cells and tissues. This diversity allows for the identification of many different biomarkers for different diseases and conditions. 3) Proteins are generally more stable than other biomolecules, such as DNA and RNA, which can be degraded by various factors, such as enzymes and environmental conditions. 4) Proteins can be readily accessed in body fluids, such as blood, urine, and saliva, making them easy to collect and analyze. 5) Some proteins are specifically expressed by cancer cells and not by healthy cells. These proteins are called cancer biomarkers to detect the presence of cancer. 6) Proteins are typically large molecules that can be detected at very low concentrations, rendering them useful for early disease diagnosis.

Proteins are essential components of cells and tissues and are involved in various biological functions, such as metabolism, signaling, and immune response. Changes in the expression or activity of specific proteins can indicate the presence or progression of a disease or condition.

Proteomic biomarkers possess potential applications in diverse healthcare areas, encompassing disease diagnosis, prognosis, disease progression monitoring, and prediction of treatment response.^[^ ^101^ ^]^ For instance, in clinical settings, prostate‐specific antigen (PSA) serves as a biomarker for prostate cancer, while cancer antigen 125 (CA‐125) is utilized as a biomarker for ovarian cancer. Additionally, proteomic biomarkers can be used to evaluate drug efficacy and toxicity, enabling more personalized and effective treatments.

**Figure 5:**
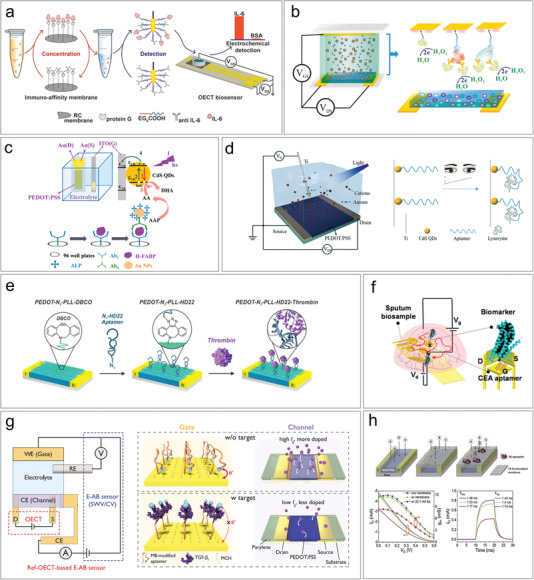
Proteomic biomarkers detection based on OECTs. a) The detection of interleukin‐6 (IL‐6) by binding with anti‐IL6. Reproduced with permission.^[^ ^104^ ^]^ Copyright 2018, Royal Society of Chemistry. b) The detection of HER2 through labeling with catalytic nanoprobes. Reproduced with permission.^[^ ^37^ ^]^ Copyright 2017, Wiley‐VCH. c) The detection of heart‐type fatty acid binding protein (H‐FABP) by OPECT through labeling with catalytic nanoprobes. Reproduced with permission.^[^ ^105^ ^]^ Copyright 2022, Elsevier. d) The detection of lysozyme by aptamer‐based OPECT. Reproduced with permission.^[^ ^107^ ^]^ Copyright 2022, Royal Society of Chemistry. e) The detection of thrombin by aptamer‐based OECT. Reproduced with permission.^[^ ^111^ ^]^ Copyright 2023, American Chemical Society. f) The detection of multi biomarker CEA, NSE, and CA125 by integrated OECT. Reproduced with permission.^[^ ^112^ ^]^ Copyright 2022, Elsevier. g) Highly sensitive detection of transforming growth factor beta 1 (TGF‐β1) by aptamer based OECT. Reproduced with permission.^[^ ^113^ ^]^ Copyright 2023, Springer Nature. h) The detection of Aβ aggregates by impeding the movement of ions from the electrolyte to the channel. Reproduced with permission.^[^ ^114^ ^]^ Copyright 2019, Elsevier.

Utilizing antigen‐antibody specific interactions is a widely employed and straightforward approach for detecting proteins in OECT sensors. Typically, gate electrodes are sequentially modified by self‐assembled monolayers (SAM) and probe antibodies/antigens layer for the specific anchoring of target proteins.^[^ ^102^, ^103^ ^]^ Gentili et al. successfully demonstrated the specific detection of interleukin‐6, a cytokine strongly associated with inflammation or disease progression, using OECTs (**Figure** **5**a).^[^ ^104^ ^]^ Specifically, the oligo(ethylene glycol) (OEG)‐terminated self‐assembled alkanethiolate monolayers were immobilized on the Au gate to bind anti interleukin 6 (anti‐IL‐6) antibodies. The interaction between IL‐6 and anti‐IL‐6 antibodies resulted in a change in the channel current. To improve the sensitivity to IL‐6, selective capturing membranes were utilized to increase the local concentration of IL‐6, achieving a limit of detection of 220 pg mL^−1^. Label‐free methods offer a cost‐effective and rapid approach for detection, while labeling strategies enhance the detection sensitivity. Fu et al. developed an ultrahigh sensitivity protein sensor for the detection of cancer biomarker human epidermal growth factor receptor 2 (HER2), as shown in Figure 5b.^[^ ^37^ ^]^ The device employs catalytic nanoprobes that are labeled with HER2 detection antibodies and horseradish peroxidase (HRP), an electrochemically active enzyme, enabling the specific detection of HER2 at a very low detection limit of 10^−14^ g mL^−1^ (10^−16^ M). The concentration of HER2 in a sample determines the amount of HRP that is immobilized on the gate electrode, which subsequently influences the redox current level on the gate electrode upon the addition of H_2_O_2_. The nanoprobes that are immobilized on the gate electrodes play a critical role in the device's response to the addition of H_2_O_2_ and the achievement of an extremely low detection limit. This labeling method was also applied in OPECT sensor for the detection of heart‐type fatty acid binding protein (H‐FABP), which is a biomarker of acute myocardial infarction.^[^ ^105^ ^]^ As shown in Figure 5c, the secondary antibody was modified by alkaline phosphatase (ALP) loaded Au nanoparticles. In the presence of ALP, the hydrolysis of ascorbic acid‐2‐phosphate (AAP) to ascorbic acid (AA) occurs rapidly, serving as a sacrificial reagent that quenches the photogenerated holes on the valence band of CdS quantum dots (CdS QDs). Organic photoelectrochemical transistor has enabled detections of multiple protein biomarkers, including C‐reactive protein,^[^ ^106^ ^]^ lysozyme,^[^ ^107^ ^]^ alkaline phosphatase,^[^ ^108^ ^]^ and prostate specific antigen (PSA).^[^ ^109^ ^]^

In addition to utilizing antibodies as probes for immobilizing proteins, aptamers can also be served as alternative recognition receptors to capture the protein analytes.^[^ ^110^ ^]^ Aptamers can bind to specific target proteins with high affinity and specificity through selective binding, where the aptamer's 3D structure allows it to recognize and bind to specific regions on the protein surface through non‐covalent interactions. Figure 5d,e demonstrate the application of aptamer‐based protein detection for the identification of lysozyme and thrombin, biomarkers for chronic dry eye syndrome and cardiovascular diseases, respectively. ^[^ ^107^, ^111^ ^]^ Zhang et al. demonstrated a multi‐channel integrated sensor for the detection of kinds biomarkers of lung cancer (Figure 5f).^[^ ^112^ ^]^ The aptamers were immobilized on gate to specifically bind with carcinoembryonic antigen (CEA), neuron‐specific enolase (NSE), and cancer Antigen 125 (CA125) biomarkers. This portable sputum device enables the continuous in‐home monitoring of biomarker level variations in lung cancer patients. Ji et al. demonstrated the higher sensitivity of OECT compared to electrochemical transistors in electrochemical aptamer‐based sensors for detecting transforming growth factor beta 1 (TGF‐β1), a crucial biomarker for wound healing (Figure 5g).^[^ ^113^ ^]^ Results shows that the sensitivity of OECT sensor is 3 and 4 orders of magnitude higher than that of bare electrochemical sensors.

Besides specific interactions with bio‐recognizers such as antibodies and aptamers, the detection of protein biomarkers can be achieved by impeding the movement of ions from the electrolyte to the channel. Wustoni et al. presented an OECT biosensor for detecting amyloid‐β, which incorporated a molecularly selective isoporous membrane between the channel and the electrolyte (Figure 5h).^[^ ^114^ ^]^ Upon the adsorption of Aβ aggregates on the membrane, the membrane pores become blocked, impeding the transport of cations from the electrolyte to the channel. This resulted in a reduction in transconductance and a slower response time. The selectivity detection of Aβ aggregates is guaranteed by the modification of Congo red molecules on the membrane, which have strong affinity to Aβ aggregates. This method does not require any chemical modification or anchoring of biomolecules on the electronic components (gate and channel), making it more tolerant to device design and allowing for long‐term device stability. Subsequently, Koklu et al. integrated a microfluidic channel with the OECT amyloid‐β sensor.^[^ ^115^ ^]^ The incorporation of microfluidics reduced the limit of detection from nanomolar to femtomolar and extended the detection range to 2.21 fM−221 nM. It was observed that the n‐type sensor exhibited superior sensitivity compared to the p‐type sensor in the study, with lower power consumption when operated at the subthreshold regime.

#### 3.1.3 Metabolic Biomarkers

Metabolic biomarkers, downstream of genes and protein networks, are a well‐studied area of research, as shown in **Table** **1**. They can be used to detect changes in metabolic activity associated with disease, exposure to toxins, or response to treatment. Metabolic biomarkers can be detected in diverse biological samples, including blood, urine, tears, sweat, and saliva. They are typically small molecules that can be detected through self‐redox reactions or enzyme sensing methods. Electroactive metabolites, such as ascorbic acid (AA) can be catalyzed by gate electrodes made of materials like platinum, which enables high detection sensitivity but limited selectivity. Inactive metabolites, such as glucose, lactic acid, and cholesterol, require enzymatic catalysis for detection.

**Table 1:** Summary of OECT‐based biomarkers detections (molecular, cells, bacteria, and virus).

Analytes	Detection limit	Corresponding disease	Bio‐reorganization sites	Ref.
**Genomic biomarkers**				
DNA	100 fM	Cancer progression and aggressiveness	Gate (Carbon)	[93]
miRNA 21	10 fM	Cancer	Gate (Au)	[95]
miRNA 21	0.12 fM	Cancer	Gate (MOFs/TiO2)	[98]
microRNA	2 pM	Human cervical cancer	Gate (Au NPs/Au)	[92]
miRNA‐17	1 pM	Cancer	Gate (CdS QDs/TiO2)	[97]
mRNA	0.01 ng mL^−1^	Cancer	Channel (PEDOT:PSS)	[94]
**Proteomic biomarkers**				
HER2	10 fg mL^−1^	Breast cancer	Gate (Au)	[37]
Caspase‐3	0.1 pM	Cancers and neurodegenerative diseases	Gate (AuNPs/Au)	[152]
PSA	10 fg mL^−1^	Prostate cancer	Gate (Au)	[102]
PSA	0.01 U L^−1^	Prostate cancer	Gate (BCP/Bi_2_S_3_/FTO)	[109]
Survivin protein	10 pg mL^−1^	Osteosarcoma	Gate (Au)	[103]
Interleukin‐6	220 pg mL^−1^	Inflammation	Gate (Au)	[104]
C‐reactive protein	1 pg mL^−1^	Inflammation	Gate (Au NCs/TiO2/CFM)	[106]
ALP	0.01 U L^−1^	Skeletal status	Electrolyte (Tris‐HCl solution)	[108]
H‐FABP	32.2 fg mL^−1^	Acute myocardial infarction	Gate (CdS QDs/ITO)	[105]
Lysozyme	550 pg mL^−1^	Chronic dry eye syndrome	Gate (CdS QDs/Ti)	[107]
CEA	1 pg mL^−1^	Lung cancer	Gate (Au)	[112]
NSE	0.1 pg mL^−1^	Lung cancer		
CA125	50 µU mL^−1^	Lung cancer / ovarian cancer		
TGF‐β1	1 ng mL^−1^	Wound healing	Gate (Au)	[113]
Thrombin	22 nM	Cardiovascular diseases	Channel (PEDOT‐N3)	[111]
Amyloid‐β	2.21 fM	Alzheimer's disease	Electrolyte (PBS)	[115]
Aβ aggregates	2.21 pM	Alzheimer's disease	Electrolyte (PBS and human serum)	[114]
Human IgG	50 fg mL^−1^	Infections	Gate (ITO)	[153]
SARS‐CoV‐2 IgG	1 fM	SARS‐CoV‐2 infections	Gate (Au)	[90]
SARS‐CoV‐2 Spike 1 protein antibody	10 fg mL^−1^	SARS‐CoV‐2 infections	Channel (PT‐COOH)	[154]
**Metabolites**				
Glucose	1 nM	Diabetes	Gate (P‐90/Au) Channel (P‐90)	[124]
Glucose	10 nM	Diabetes	Gate (P‐90/Au) Channel (P‐90)	[125]
Glucose	30 nM	Diabetes	Gate (Pt)	[43]
Glucose	100 nM	Diabetes	Gate (Pt NPs/Au)	[116]
Lactate	1 µM	Sepsis and shock	Gate (Pt NPs/Au)	
Lactate	10 µM	Primary tumors	Gate (PEDOT:PSS/Au)	[155]
Lactic acid	10 µM	Resuscitation	Gate (P‐90/Au) Channel (P‐90)	[123]
Lactic acid	1 nM	Predicting multiple organ failure	Gate (Pt)	[117]
Ascorbic acid	10 nM	Scurvy	Gate (Au)	[128]
Sarcosine	50 nM	Prostate cancer	Gate (AAO/Pt)	[119]
Sialic acid	8 µM	Cancer	Gate (PABA/glass carbon)	[156]
Sialic acid	1 nM	Cancer	Gate (Silver)	[118]
Sialic acid	0.1 mM	Cancer	Gate (MWCNTs/Au)	[126]
Uric acid	4.5 µM	Gout, Inflammation	Gate (Pt/C)	[157]
Uric acid	100 nM	Gout, Inflammation	Gate (Pt)	[43]
Uric acid	1 nM	Gout, Inflammation	Gate (PEDOT/carbon fiber)	[158]
Urea	1 nM	Kidney Disease	Gate (PANI/MWCNT/cotton)	[159]
Urea	195 nM	Kidney Disease	Gate (Pdots/PTAA/ITO)	[160]
Cholesterol	50 µM	Cardiovascular diseases	Gate (PEDOT:PSS/PVA/Au)	[130]
Cholesterol	100 nM	Cardiovascular diseases	Gate (PANI/Nafion‐graphene/Pt)	[122]
**Hormones**				
Cortisol	100 pM	Mental health	Gate (Au)	[138]
Cortisol	8.8 ag mL^−1^	Mental health	Channel (PEDOT:PSS)	[39]
Cortisol	10 nM	Mental health	Channel (PEDOT:PSS)	[139]
Epinephrine	90 pM	Mental health	Gate (Au)	[135]
Epinephrine	1 µM	Mental health	Gate (Pt)	[136]
Epinephrine	0.1 nM	Mental health	Gate (SWNTs/Pt)	[137]
**Neurotransmitters**				
Dopamine	1 nM	Nervous system disorder	Gate (Pt)	[131]
Dopamine	5 nM	Nervous system disorder	Gate (Carbon fiber)	[87]
Dopamine	30 nM	Nervous system disorder	Gate (Pt)	[38]
Dopamine	30 nM	Nervous system disorder	Gate (Pt)	[43]
Dopamine	1 nM	Nervous system disorder	Gate (PPy/NFs/PA6)	[132]
Dopamine	37 nM	Nervous system disorder	Channel (AuNPs/PEDOT:PSS)	[161]
Acetylcholine	5 µM	Nervous system disorder Alzheimer's disease	Channel (PEDOT‐PAH)	[133]
Nitric oxide	3 nM	Chondrocyte cell death and osteoarthritis	Gate (Poly‐5A1N/ polyimide)	[162]
**Cells**				
T98G cell	Single cell	Cancer	Channel (PEDOT:PSS)	[41]
MCF‐7 cells	10 cells µL^−1^	Human breast cancer	Gate (Au)	[142]
Caco‐2; NPC43	/	Cancer	Channel (PEDOT:PSS)	[68]
NPC43	/	Cancer	Channel (PEDOT:PSS)	[141]
**Bacteria**				
Escherichia coli	10^3^ cfu mL^−1^	Gastrointestinal disease	Channel (PEDOT:PSS)	[148]
Salmonella Enteritidis	/	Typhoid Fever	Channel (PEDOT:PSS)	[163]
**Virus**				
H1N1	0.025 HAU	Human influenza	Channel (2,6‐Sialyllactose‐grafted PEDOT)	[71]
H1N1	0.015 HAU	Human influenza	Channel (2,6‐Sialyllactose‐grafted PEDOT)	[91]
SARS‐CoV‐2 spike protein	1 fg mL^−1^	SARS‐CoV‐2 infections	Gate (Au)	[149]
SARS‐CoV‐2 spike protein	1.2 zM	SARS‐CoV‐2 infections	Gate (Au)	[150]
SARS‐CoV‐2 Virus	/	SARS‐CoV‐2 infections	Gate (Au)	
MERS‐CoV Spike protein	0.57 aM	SARS‐CoV‐2 infections	Gate (Au)	
SARS‐CoV‐2 RBD	26 zM	SARS‐CoV‐2 infections	Channel (PEDOT:PSS)	[151]
SARS‐CoV‐2 virus	26 TCID_50_ mL^−1^	SARS‐CoV‐2 infections		
SARS‐CoV‐2 spike protein	100 aM	SARS‐CoV‐2 infections	Gate (Au)	[164]

**Figure 6:**
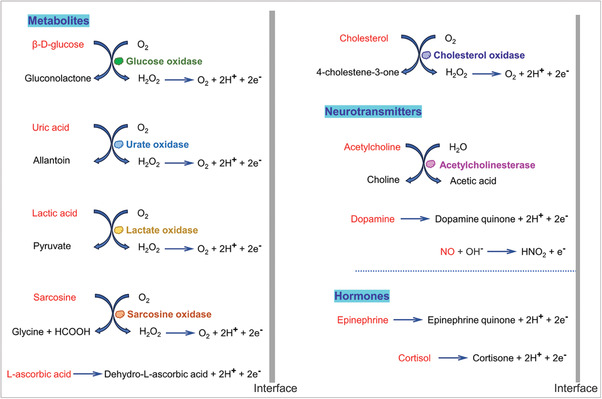
Redox reactions of metabolites, neurotransmitters, and hormones.

As one of the standard physical examination parameters, glucose concentration is strongly linked to diabetes, liver disease, and cardiovascular diseases. Glucose can be catalyzed by glucose oxidase (GOx) and produces hydrogen peroxide and gluconic acid (**Figure** **6**). The hydrogen peroxide can be further catalyzed by a Pt electrode, resulting in the transfer of electrons from the metabolite to the gate electrode and causing a potential drop of the gate electrode. The effective gate voltage (*V_G_^eff^*) of OECT can be expressed by:^[^ ^88^ ^]^

(2) $$V_{G}^{eff}=V_{G}\;+\left(1+\gamma \right)\frac{kT}{2q}\ln\left[H_{2}O_{2}\right]+Constant$$

where *γ* is the ratio between the capacitances of electrolyte/channel interface *C_c_* and electrolyte/gate interface *C_G_*, *k* is the Boltzmann constant, and *T* is the temperature. Metabolites such as uric acid, lactate, and sialic acid have similar sensing mechanisms, as exemplified by glucose detection.

**Figure 7:**
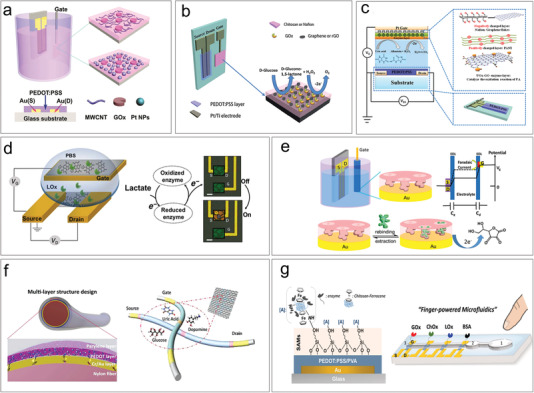
Metabolic biomarkers detection based on OECTs. a) Enzyme‐based glucose detection with gate modified by multi‐walled carbon nanotubes (MWCNT) and Pt NPs. Reproduced with permission.^[^ ^120^ ^]^ Copyright 2011, Wiley‐VCH. b) Enzyme‐based glucose detection with gate modified by graphene. Reproduced with permission.^[^ ^121^ ^]^ Copyright 2013, Royal Society of Chemistry. c) Multi metabolites detection with gate modified by PANI/Nafion‐graphene bilayer film. Reproduced with permission.^[^ ^122^ ^]^ Copyright 2015, Wiley‐VCH. d) Enzyme‐based lactate detection using n‐type OECT. Reproduced with permission.^[^ ^123^ ^]^ Copyright 2018, AAAS. e) The detection of ascorbic acid using MIP‐based OECT. Reproduced with permission.^[^ ^128^ ^]^ Copyright 2018, Elsevier. f) Fiber‐based OECT sensor for the detection of multi metabolite biomarkers. Reproduced with permission.^[^ ^43^ ^]^ Copyright 2018, Wiley‐VCH. g) OECT microarrays integrated with finger‐powered microfluidics for the detection of lactate, glucose, and cholesterol in blood and saliva samples. Reproduced with permission.^[^ ^130^ ^]^ Copyright 2016, Wiley‐VCH.

The two primary indicators of interest in metabolic testing are detection sensitivity and selectivity. Typically, modification of nanomaterials on biorecognition sites such as gate electrode can enhance sensitivity.^[^ ^116^, ^117^, ^118^, ^119^ ^]^ The use of nanomaterials increases the electrode's surface area‐to‐volume ratio and conductivity, enlarges the contact area between the analyte and electrode, and ultimately promotes charge transfer between the electrode and analyte. Additionally, some nanoparticles such as platinum and gold nanoparticles exhibit electrocatalytic effects that can facilitate the redox reactions of the analyte. For instance, when platinum nanoparticles are used to modify the Pt gate electrode, their superior catalytic activity toward hydrogen peroxide can lower the detection limit of glucose to 5 nM.^[^ ^120^ ^]^ This is three orders of magnitude lower than the detection limit of the device without nanoparticles (**Figure** **7**a). Nanomaterials such as graphene and reduced graphene oxide can also improve the sensitivity of the device and extend its detection range. This is because they can increase the surface‐to‐volume ratio of the gate electrode and enhance charge transfer between the analytes and electrodes. (Figure 7b).^[^ ^121^ ^]^

A challenge in metabolic testing is selectivity, as human bodily fluids contain multiple metabolites, including glucose, uric acid, lactate, and others, which may interfere with each other during their detections. Chitosan and Nafion can facilitate the immobilization of GOx onto the gate electrode and improve the selectivity of the device, demonstrating excellent ion selectivity and biocompatibility. Liao et al. have demonstrated another highly selective enzyme biosensor by modifying a polyaniline (PANI)/Nafion‐graphene bilayer film on a Pt gate electrode (Figure 7c).^[^ ^122^ ^]^ The PANI film is in the H^+^ protonated emeraldine salt form, which can repel positively charged molecules, such as dopamine, via electrostatic forces. In contrast, the negatively charged Nafion contains acidic sulfonic groups in its Teflon backbone, which can impede the diffusion of anionic electroactive substances, such as ascorbic acid and uric acid. Graphene flakes are used to improve the electrocatalytic activity and conductivity of the gate electrode. As a result, the PANI/Nafion‐graphene bilayer film can block the diffusion of both positively and negatively charged molecules to the Pt gate and improve the selectivity of the device for H_2_O_2_. This sensor can achieve highly selective and sensitive detection of H_2_O_2_, uric acid, cholesterol, and glucose in saliva.

The enzyme‐based electrocatalysis was proven to be more efficient in electron‐transporting (n‐type) OECTs.^[^ ^123^, ^124^, ^125^ ^]^ N‐type polymer P‐90 is an active material is based on NDI‐T2 copolymer, which have side chains full of polar glycol groups. The hydrophilic side chains provide polar groups for enzyme modification, and promote the ion transport and injection. Electrons generated from the enzymatic reaction could be directly transferred to the backbone of P‐90 and increase its conductivity. The P‐90 based n‐type OECTs were used for the detection of lactate and glucose, which shows high sensitivity and selectivity (Figure 7d).^[^ ^123^, ^124^, ^125^ ^]^

In addition to enzyme‐based sensing, metabolites can also be detected using methods such as covalent immobilization and molecular imprinting. For example, 3‐aminophenylboronic acid (APBA) can specifically bind to sialic acid, which is a cancer biomarker, through a covalent bond.^[^ ^126^ ^]^ When sialic acid is immobilized on a gate electrode, the electron transfer impedance increases significantly due to the formation of a more hydrophobic gate/electrolyte interface. Molecular imprinting technology is a powerful technique used to create highly selective molecular recognition sites that find applications in various fields such as analytical chemistry, sensors, and drug delivery.^[^ ^127^ ^]^ Zhang et al. demonstrated the use of a molecular imprinted polymer (MIP)‐based OECT biosensor for the detection of ascorbic acid (AA).^[^ ^128^ ^]^ The AA MIP film was fabricated on an Au gate electrode using electrochemical polymerization (Figure 7e). The MIP‐modified OECT gate exhibited a detection limit of 10 nM, which is three orders of magnitude lower than that of the device without molecularly imprinted polymer.

It has been demonstrated that OECT biosensors can be used for the detection of multiple metabolites in bodily fluids. The combination of OECT biosensors with fabric substrates and microfluidics can bring the detection closer to practical applications. Yang et al. demonstrated a fabric OECT for the detection of glucose, uric acid and dopamine (Figure 7f).^[^ ^43^ ^]^ The devices are fabricated on nylon fibers by coating Au and PEDOT:PSS layers, and then be weaved with PVA protecting yarns. Flexible fabric biosensors were integrated into a diaper, which can be remotely controlled by a mobile phone for monitoring glucose concentration of urine. Multianalyte sensing platforms enable simultaneous detection of multiple metabolites, providing a comprehensive analysis of a sample in a single assay. This approach saves time and resources, and reduces the risk of false positives or negatives that may occur when using multiple single‐analyte assays. Microfluidics can reduce sample and reagent consumption, lowering the cost of analysis and minimizing waste. The high surface‐to‐volume ratio of microfluidic devices enables efficient mass transport and reaction kinetics, providing rapid and high‐throughput analysis. By precisely controlling fluid and surface interactions, microfluidic devices can achieve high sensitivity and selectivity, allowing for efficient capture, separation, and detection of biomolecules.^[^ ^124^, ^129^ ^]^ These devices can be easily integrated with other analytical components, such as sensors, pumps, and valves, to create fully automated systems that can perform complex analytical tasks without manual intervention. Pappa et al. demonstrated OECT microarrays integrated with finger‐powered microfluidics for the detection of lactate, glucose, and cholesterol in blood and saliva samples. This portable and easy‐to‐handle platform (Figure 7g) enables the application of other types of enzymatic biosensors toward accurate, rapid, non‐invasive healthcare monitoring.^[^ ^130^ ^]^

#### 3.1.4 Hormones and Neurotransmitters Biomarkers

Hormones and neurotransmitters are chemical messengers that have crucial roles in intercellular communication. While many of them, such as epinephrine, cortisol, norepinephrine, and dopamine exhibit electroactivity, as shown in Figure 4.^[^ ^38^, ^131^ ^]^ The principle of hormone and neurotransmitter detection is similar to that of metabolite detection. Analytes with electroactivity can be directly oxidized and release electrons, while analytes without electrochemical activity can be detected through methods such as immune reactions, covalent bonding, and molecular imprinting.

**Figure 8:**
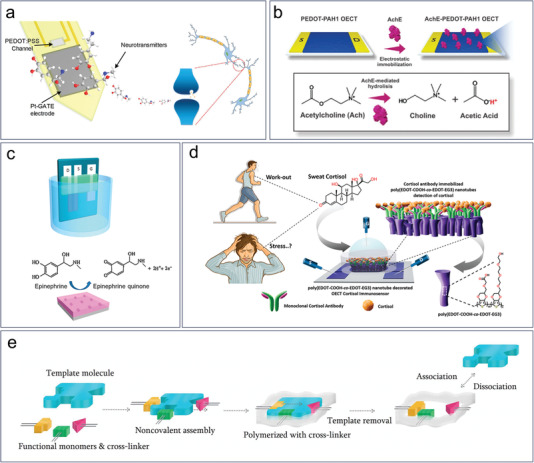
Hormones and neurotransmitters biomarkers. a) Real‐time measurement of dopamine in rat brain by a micro probe‐shaped OECT device. Reproduced with permission.^[^ ^38^ ^]^ Copyright 2020, eLife Sciences Publications, Ltd. b) The detection of acetylcholine using enzyme‐based OECT. Reproduced with permission.^[^ ^133^ ^]^ Copyright 2021, Wiley‐VCH. c) A highly sensitive epinephrine sensor with gate modified by nano materials. Reproduced with permission.^[^ ^137^ ^]^ Copyright 2015, Royal Society of Chemistry. d) A cortisol sensor with OECT channel decorated by polymer nanotubes. Reproduced with permission.^[^ ^39^ ^]^ Copyright 2022, American Chemical Society. e) A MIP‐based OECT sensor for the detection of cortisol. Reproduced with permission.^[^ ^139^ ^]^ Copyright 2018, AAAS.

Dopamine is a neurotransmitter that is primarily synthesized in the human brain and is essential for facilitating impulse transmission between cells. Upon catalysis by a Pt electrode, dopamine undergoes oxidation to form quinone and release electrons, which are then transferred to the gate electrode, resulting in variations in the channel current.^[^ ^43^, ^87^, ^131^, ^132^ ^]^ Xie et al. conducted a study demonstrating the real‐time measurement of dopamine released from living rats (**Figure** **8**a).^[^ ^38^ ^]^ Microarray of OECTs were fabricated on a micro probe‐shaped polyethylene terephthalate substrate. The probe with multi OECTs were inserted into rat brain and covered brain locations including ventral tegmental area, nucleus accumbens and different parts of caudate putamen. The dopamine released in different regions of the brain was monitored in response to neural stimulations. This work marks the first time that transistor arrays have been used to measure neurotransmitter release in a living brain. Neurotransmitters such as acetylcholine can be detected by utilizing enzymes since they cannot be directly catalyzed by electrodes. Fenoy et al. demonstrated a OECT using PEDOT and polyallylamine hydrochloride (PAH) composites as channel material (Figure 8b).^[^ ^133^ ^]^ The incorporation of PAH not only improve the device electrical performances, but also proves side chains for anchoring of acetylcholinesterase (AchE). Acetylcholine can be effectively catalyzed by AchE and produces choline and acetic acid, which decreases the local pH value and changes the threshold voltage of OECTs. In addition to enzymatic reactions, Jang et al. presented a study showcasing a sensitive and highly selective detection of acetylcholine, without relying on enzymatic reactions. They achieved this by employing a synthetic receptor to capture acetylcholine, thereby demonstrating the feasibility of an alternative approach for developing acetylcholine sensors based on OECTs.^[^ ^134^ ^]^

Hormones exert a substantial influence on health despite their relatively low concentrations within the human body, and imbalances can contribute to a range of diseases. Electroactive hormones such as epinephrine and melatonin can be directly detected by redox reactions at a Pt gate electrode (Figure 8c). ^[^ ^135^, ^136^ ^]^ The sensitivity of an epinephrine sensor can be improved by modifying graphene flakes, graphene oxide , and single‐walled carbon nanotubes.^[^ ^137^ ^]^ Furthermore, by modifying a Nafion film on the Pt gate, the selectivity of the devices can be enhanced, leading to an impressive detection limit of 0.1 nM.^[^ ^137^ ^]^ Cortisol, an adrenocorticosteroid stress hormone, can be detected using OECTs that combine immunoassays and molecular imprinting. ^[^ ^39^, ^138^, ^139^ ^]^ Janardhanan et al. demonstrated a cortisol sensor with OECT channel as the recognition site.^[^ ^39^ ^]^ A layer of poly(EDOT‐COOH‐co‐EDOT‐EG3) nanotubes, fabricated by electrochemical polymerization, was decorated onto a PEDOT:PSS film, allowing for anti‐cortisol antibodies to conjugate with its side chains (Figure 8d). It was found that devices with a nanostructured channel showed higher sensitivity, with a detection range from 1 fg mL^−1^ to 1 µg mL^−1^, and an excellent limit of detection of 0.0088 fg mL^−1^. Parlak et al. developed an artificial recognition membrane based on molecularly imprinted polymers (MIPs) that was covered onto a PEDOT:PSS channel film (Figure 8e).^[^ ^139^ ^]^ This membrane is capable of selectively controlling the transport of cortisol from sweat to the channel film. The detection mechanism is based on the fact that when cortisol is present, the membrane pores that allow ion transport from the electrolyte to the channel are blocked. As a result, the modulation of OECT is weakened, leading to a change in the channel current. The artificial MIP membrane is stable, robust, and easily integrated, making it highly suitable for a wide range of applications in biosensing.

### 3.2 Cell Biomarkers

Cell characteristics, including quantity, dimensions, morphology, and barrier integrity, serve as important biomarkers that provide essential insights into the overall health and functionality of an organism. For example, the quantity of leukocytes correlates with bacterial or viral infections, while the quantity of erythrocytes and the mean corpuscular volume can be employed in the diagnosis of anemia, establishing them as valuable diagnostic biomarkers.

**Figure 9:**
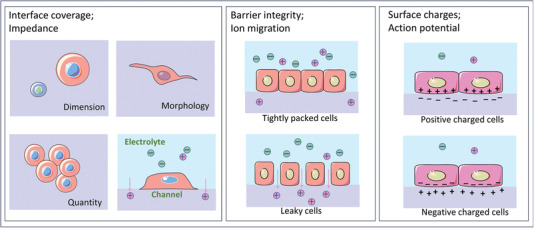
Working principles of OECT‐based cell biomarker detection.

The mechanism of cell detection is more complex compared to the detection of biomolecules, as shown in **Figure** **9**. From the current research, the changes in electrical signals caused by cells are primarily attributed to impedance. The magnitude of impedance is collectively determined by factors such as cell dimensions, morphology, and quantity. Additionally, OECTs can be utilized to assess the integrity of epithelial cells, thereby enabling the detection of tissue pathologies. Intact and tightly arranged epithelial cells can impede the transport of ions between the electrolyte and the channel, thereby suppressing the modulation of the transistor. Moreover, charged cells can influence the concentration of carriers in the channel, thereby altering the magnitude of channel current. For example, excitation of cardiac muscle cells can generate action potentials, which affect the distribution of channel charge carriers and lead to changes in transistor performance.^[^ ^140^ ^]^

**Figure 10:**
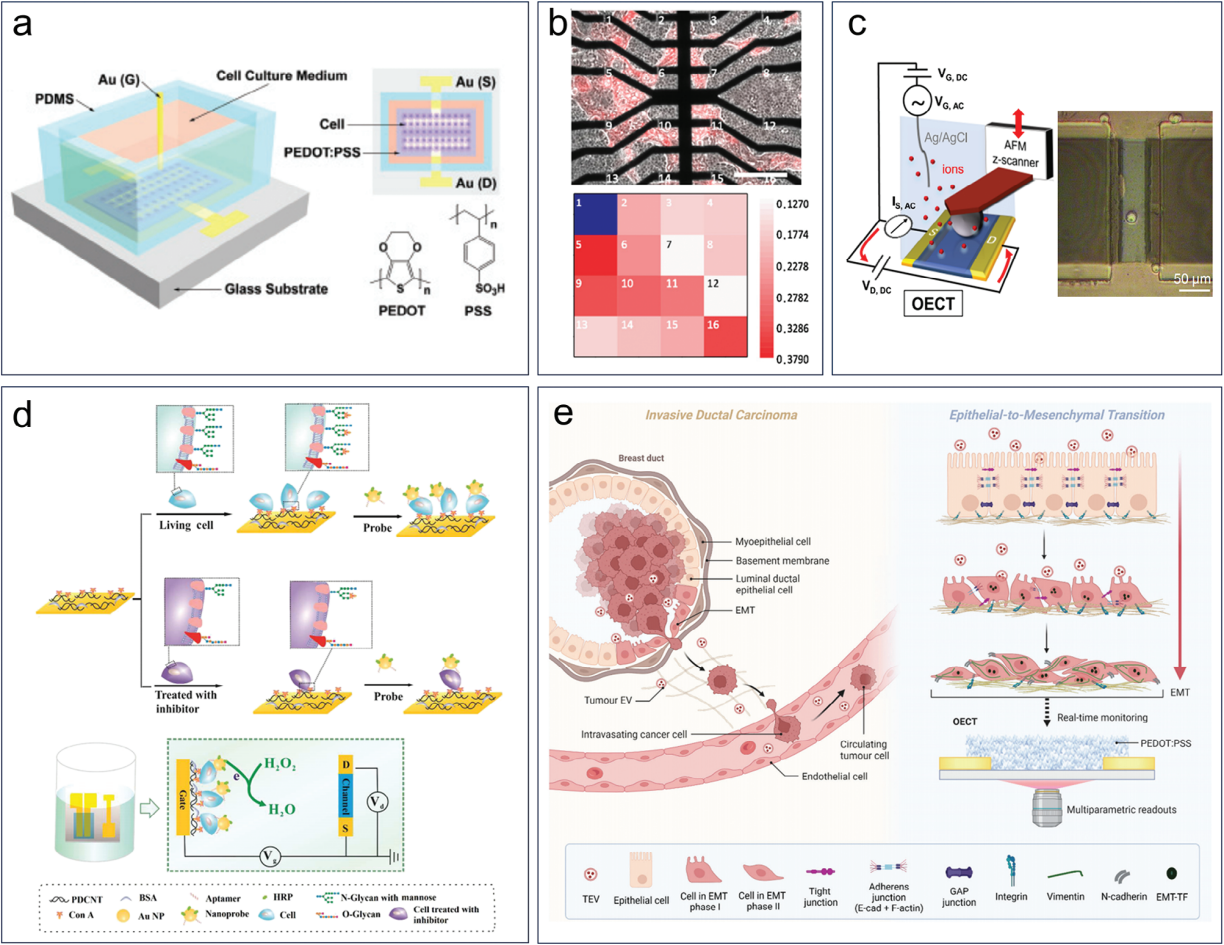
Cell biomarkers detection based on OECTs. a) Monitoring of cell activities and morphology by OECTs.^[^ ^66^ ^]^ Copyright 2010, Wiley‐VCH. b) Detection of the cancer cells invasion to normal cells using OECTs. Reproduced with permission.^[^ ^141^ ^]^ Copyright 2019, Elsevier. c) Impedance‐based single‐cell detection. Reproduced with permission.^[^ ^41^ ^]^ Copyright 2022, Springer Nature. d) Sensitive human breast cancer cells detection. Reproduced with permission.^[^ ^142^ ^]^ Copyright 2018, American Chemical Society. e) Monitoring of epithelial‐to‐mesenchymal transition caused by tumor‐derived extracellular vesicles . Reproduced with permission.^[^ ^143^ ^]^ Copyright 2023, Wiley‐VCH.

Both the gate electrode and channel of OECTs can serve as biorecognition sites in cell biosensors, onto which cells can physically adsorb. Lin et al. cultivate cancer cell lines KYSE30 and fibroblast cell lines (HFF1) on the PEDOT:PSS channel, which shows excellent biocompatibility for cell growth (**Figure** **10**a).^[^ ^66^ ^]^ Owing to the larger coverage area of round‐shaped cancer cell lines compared to fibroblast cell lines, a greater change in gate voltage can be detected upon detachment from the PEDOT:PSS film. This is the first reported application of OECTs for cell detection, demonstrating their potential for in vitro monitoring of cell activities and morphology. The destruction of epithelial cells is often considered a hallmark of malignant tumors. Yeung et al. investigated the effects of invasive cancer cells on a normal epithelial monolayer using OECTs (Figure 10b).^[^ ^141^ ^]^ The nasopharyngeal cancer cell line NPC43 can be distinguished from other epithelial or cancer cell types, and its invasion to the epithelial Madin‐ Darby Canine Kidney cells can be spatial mapped by 16‐channel OECT array. The aforementioned study indicates that the morphology and packing density of cancer cells can affect the electrical characteristics of OECTs, thereby reflecting the biological information.

In addition to investigating the effects of cancer cell morphology on the electrical properties of OECTs, research has advanced to the point where the number of cells can be accurately studied at the single‐cell level (Figure 10c).^[^ ^41^ ^]^ To optimize detection sensitivity, a mathematical model was first developed to predict the dependence of sensitivity on frequency, sensor geometry, and semiconductor material properties. The optimized results were then applied to cell detection using OECTs. The sensor size was reduced to match the dimensions of individual cells, enabling impedance‐based single‐cell detection. Ultimately, single‐cell detection of the human malignant glioma cell line T98G was achieved, and a high current signal gain of 20.2 dB was demonstrated. In addition to adhering directly to the organic channel film, which is typically PEDOT:PSS, cancer cells can also be selectively immobilized on gate electrodes (Figure 10d). By integrating nanoprobes, OECTs were able to detect human breast cancer cells (MCF‐7) at concentrations as low as 10 cells µL^−1^, and detect changes in the expression of mannose on the cell surface.^[^ ^142^ ^]^ Recently, Traberg et al. demonstrated a noninvasive, real‐time detection of tumor‐derived extracellular vesicles induced epithelial‐to‐mesenchymal transition using OECTs (Figure 10e).^[^ ^143^ ^]^ The detection mechanism of this system exhibits similarities to previously documented cell sensors.^[^ ^68^, ^144^, ^145^, ^146^, ^147^ ^]^ Specifically, when normal cells undergo malignant transformation into cancer cells, there is an observable alteration in their cellular morphology. The tightly packed arrangement of cells on the channel becomes more relaxed, leading to a reduction in cellular barrier integrity and a consequent decrease in impedance. As a result, the process of ion doping becomes more facilitated.

### 3.3 Bacterial and Virus as Biomarkers

Given that bacteria and viruses are classified as pathogens and their detection and analysis commonly involve nucleic acid and protein testing, these two entities can be categorized together. Bacterial or viral infections can be preliminarily diagnosed through blood tests, which involve assessing biomarkers such as white blood cell count, C‐reactive protein levels, and platelet count. However, the identification of the pathogen requires the use of specific detection methods such as genetic sequencing or immunological tests. Bacterial infections commonly observed in clinical settings include Escherichia coli, staphylococcus aureus, and salmonella, while common viral infections include influenza virus, norovirus, and coronavirus. The detection of pathogens typically involves the specific interactions between proteins, and the detection methods can be analogous to those used for protein biomarker detection. These infectious agents can often be detected using immunoassays that rely on the specific binding of pathogen‐derived antigens to corresponding antibodies.

**Figure 11:**
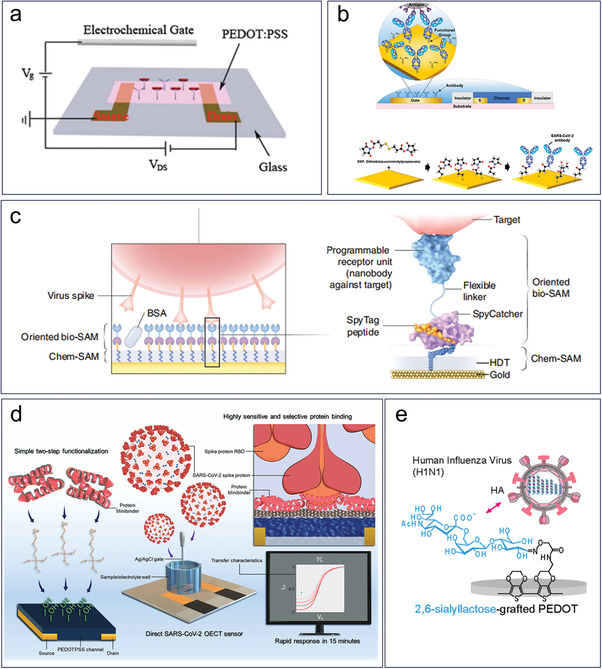
Virus and bacteria biomarkers detection based on OECTs. a) Specific detection of Escherichia coli using OECTs. Reproduced with permission.^[^ ^148^ ^]^ Copyright 2012, Royal Society of Chemistry. b) Detection of SARS‐CoV‐2 by the anchoring of antibodies. Reproduced with permission.^[^ ^149^ ^]^ Copyright 2023, Royal Society of Chemistry. c) Detection of SARS‐CoV‐2 by the anchoring of nanobodies. Reproduced with permission.^[^ ^150^ ^]^ Copyright 2021, Springer Nature. d) Detection of SARS‐CoV‐2 by the anchoring of a protein minibinder. Reproduced with permission.^[^ ^151^ ^]^ Copyright 2023, Wiley‐VCH. e) Detection of human influenza A virus by the anchoring of 2,6‐sialyllactose side chain of the electrodeposited conducting copolymer. Reproduced with permission.^[^ ^91^ ^]^ Copyright 2017, American Chemical Society.

The general approaches for detecting antigens are similar in principle. Typically, a chemical self‐assembled monolayer (SAM) is first modified at the interface to covalently bind another layer of biomolecules, which is composed of specific antibodies or proteins, to specifically capture the target analytes. For example, in a OECT based *Escherichia coli* (*E. coli*) sensor, PEDOT:PSS active layers was first grafted by silane to bind with anti‐E. coli antibodies (**Figure** **11**a).^[^ ^148^ ^]^ The detection was realized by specific capture of E. coli, with a detection limit of 10^3^ cfu mL^−1^.

The two‐step functionalization of chemical SAMs and biological SAMs at bio‐recognition sites is a common strategy used in the development of OECT‐based virus sensors. ^[^ ^149^, ^150^, ^151^ ^]^ For instance, Fan et al. conducted a study where they introduced the modification of 3,3′‐dithiodipropionic acid di(N‐hydroxy succinimide ester) (DSP) on the gold gate of an OECT.^[^ ^149^ ^]^ This modification enabled the covalent binding of anti‐severe acute respiratory syndrome coronavirus 2 (SARS‐CoV‐2) spike S1 antibodies, specifically facilitating the capture of SARS‐CoV‐2 spike protein and intact virions in saliva samples (Figure 11b). In addition to using antibody specificity for viral recognition, certain proteins that can specifically identify viruses can also be utilized as a bio‐SAM layer. Guo et al. reported a nanobody‐functionalized OECT for single molecule detection of specific antigens (Figure 11c).^[^ ^150^ ^]^ The bio‐SAM is high‐density and orientation‐controlled bioconjugation of nanobody–Spy Catcher fusion proteins, which improved the density and robustness of the biorecognition layer. The specificity of this detection platform is promised by the specific nanobody, which structure can be designed to satisfy different types of virus. The final test results are impressive, as the test has a high sensitivity, with the ability to detect single‐molecule SARS‐CoV‐2 spike protein and a detection limit for Middle East respiratory syndrome coronavirus (MERS‐CoV) spike protein of 0.57 aM. Furthermore, the test can accurately identify individuals who have been infected with the coronavirus. Another novel OECT virus sensor was developed by immobilizing a stable and cost‐effective protein minibinder on channel to specially detect the SARS‐CoV‐2 virus at concentrations as low as 40 virus particles per mL (Figure 11d).^[^ ^151^ ^]^ Moreover, some earlier studies have indicated that specific virus detection could be achieved without the use of a bio‐SAM layer.^[^ ^71^, ^91^ ^]^ Hai et al. developed trisaccharide‐grafted PEDOT for label‐free detection of the human influenza A virus (H1N1) (Figure 11e).^[^ ^91^ ^]^ The 2,6‐sialyllactose side chain of the electrodeposited conducting copolymer films can specifically interact with hemagglutinin in the envelope of H1N1, enabling sensitive detection.

### 3.4 Biophysical Biomarkers

Biophysical biomarkers are measurable physical properties that provide information about biological processes and functions in living organisms. These biomarkers can include a wide range of parameters, such as blood pressure, heart rate, body temperature, respiratory rate, blood oxygen saturation, electrophysiology signals, and more. Biophysical biomarkers can be used to monitor and diagnose a variety of medical conditions, including cardiovascular disease, diabetes, respiratory disorders, and neurological disorders. For example, blood pressure and heart rate can be used to diagnose hypertension and other cardiovascular conditions, while respiratory rate can provide information about lung function and respiratory disorders.

**Figure 12:**
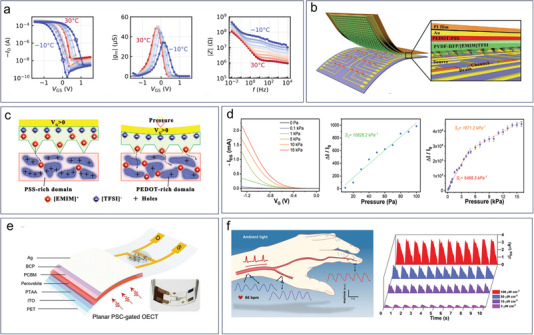
Basic biophysical biomarkers detection based on OECTs. a) OECT based temperature sensor. Reproduced with permission.^[^ ^165^ ^]^ Copyright 2022 IEEE. b) The structure of OECT based pressure sensor. c) The work mechanism of OECT based pressure sensor that employs a solid electrolyte with micro‐pyramid array's structure. d) High sensitivity of OECT based pressure sensor. b–d) Reproduced with permission.^[^ ^85^ ^]^ Copyright 2020, Wiley‐VCH. e) An OECT driven by perovskite solar cells. f) The photoplethysmogram signals monitoring under ambient light conditions. e,f) Reproduced with permission.^[^ ^168^ ^]^ Copyright 2023, Wiley‐VCH.

While the majority of OECT biosensors have traditionally focused on detecting molecular and cellular biomarkers, there has been a recent shift toward the detection of electrophysiological signals. Despite the evolving trend, there is still a shortage of biosensors that can effectively detect fundamental biophysical signals such as body temperature and blood pressure. In 2022, Bongartz et al. demonstrated for the first time the ability of OECTs to measure temperature dependence.^[^ ^165^ ^]^ They found that the transfer curves of OECTs behaved differently at temperatures ranging from −10 to 30 °C (**Figure** **12**a). The results showed that an increase in temperature led to a decrease in threshold voltage and an increase in transconductance. However, the temperature steps used in this study were too large to allow for accurate testing of human body temperatures. Further research is needed to enable OECTs to achieve precise temperature measurements and to be applied for human body temperature monitoring. Although an OECT‐based blood pressure sensor is not yet available, there are several OECT‐based pressure sensors that represent preliminary steps toward blood pressure detection. The OECT‐based pressure sensor employs solid electrolytes or ion gels, which are typically patterned as micro‐pyramid arrays using a silicon mold.^[^ ^85^, ^166^, ^167^ ^]^ The pyramid‐patterned electrolyte was positioned between the gate and channel (Figure 12b).^[^ ^85^ ^]^ In the absence of applied pressure, the electrolyte‐channel polymer contact area is minimal, resulting in insufficient driving of the channel current by the gate voltage. However, upon application of pressure, ion conduction from the electrolyte to the channel is facilitated, enabling gate modulation of the source‐drain current (Figure 12c). The OECT‐based pressure sensor can achieve high sensitivity of ≈10 000 kPa^−1^ and a low limit of detection of 1.1 Pa, enabling detection of the weight of a flower or a grain of rice (Figure 12d).^[^ ^85^ ^]^ The micro‐pyramid arrays can be utilized not only in the electrolyte, but also in the channel film and the gate, offering flexible design options. Blood oxygen saturation, also known as SpO2, is a critical biomarker for patients with respiratory or cardiovascular conditions, measuring the amount of oxygen carried by hemoglobin in the blood. Song et al. have demonstrated an OECT driven by perovskite solar cells that exhibits high transconductance and fast transient response (Figure 12e).^[^ ^168^ ^]^ The device can remotely monitor photoplethysmogram signals under ambient light conditions, including outdoor sunlight and indoor light (Figure 12f). In addition, the device can measure SpO2 under ambient light by integrating optical filters in green and red.

Electrophysiological signals are bioelectric signals generated by ionic movements across excitable cell membranes, including neurons and muscle cells. These signals are crucial for investigating the functional properties of the nervous system and the heart, and can be recorded using skin surface or implanted electrodes. Electrophysiological signals comprise diverse biomedical techniques, such as electrocardiography (ECG), electroencephalography (EEG), electromyography (EMG), electrooculography (EOG), and in vivo electrocorticography (ECoG). The frequencies of electrophysiological signals range from sub‐Hertz to ≈10 kHz, and their dynamic amplitude can vary from 1 µV to over 10 mV. The tiny change of skin potential can be locally amplified by OECTs and displayed superior signal‐to‐noise ratio compared with surface electrodes. In order to ensure the more accurate detection of electrophysiological signals, OECTs are expected to have high transconductance and fast response time.

**Figure 13:**
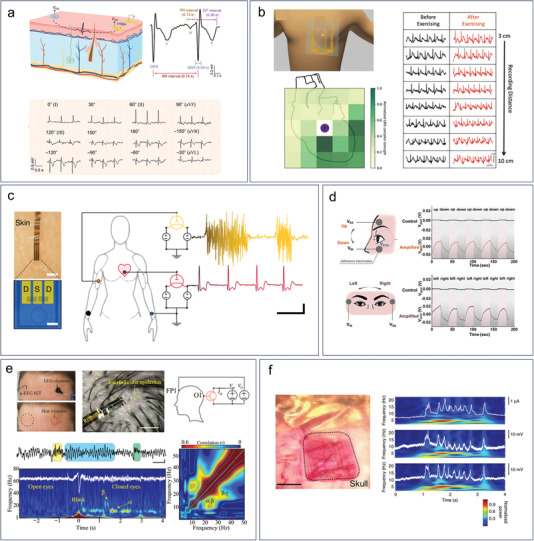
Electrophysiological signals detection based on OECTs. a) ECG signals mapping along different directions of heart. Reproduced with permission.^[^ ^169^ ^]^ Copyright 2023, AAAS. b) ECG monitoring before and after exercise. Reproduced with permission.^[^ ^170^ ^]^ Copyright 2023, Wiley‐VCH. c) Non‐invasive EMG detection with high signal noise ratio. Reproduced with permission.^[^ ^56^ ^]^ Copyright 2020, Springer Nature. d) EOG signals recorded by high gain OECT based complementary inverter. Reproduced with permission.^[^ ^171^ ^]^ Copyright 2021 National Academy of Science. e) Micrometer‐scale ion‐gated OECTs for EEG recording. Reproduced with permission.^[^ ^172^ ^]^ Copyright 2019, AAAS. f) In vivo ECoG monitoring using ultra‐thin OECTs patched onto the somatosensory cortex of rats. Reproduced with permission.^[^ ^173^ ^]^ Copyright 2013, Springer Nature.

Song et al. demonstrated an electrochemical transistors with high transconductance and fast response by introducing the highly oriented 2D conjugated metal organic frameworks (MOF) as the channel material.^[^ ^169^ ^]^ The high‐porosity crystal structure of MOF promotes the ion diffusion in the bulk across nanopores, leading to a high volumetric capacitance. MOF based electrochemical transistor arrays were fabricated on ultrathin polyimide substrates, with 12 devices distributed around the heart to record ECG signals along different directions (**Figure** **13**a). The detection result of this portable and lightweight device is comparable to the waveforms obtained from a conventional ECG testing facility. Another study was conducted to investigate the impact of physical activity on ECG monitoring using wearable OECT devices placed at various distances along the vertical axis of the heart (Figure 13b).^[^ ^170^ ^]^ The location of the heart region with the highest QRS complex amplitude changed after exercise.

Electromyography (EMG) is a technique used to record and analyze the electrical activity of muscles. EMG is widely used in clinical and research settings to evaluate the function of the neuromuscular system, including the diagnosis of muscle and nerve disorders such as muscular dystrophy, peripheral neuropathy, and myasthenia gravis. The research conducted by Cea et al. showed the successful development of an enhancement‐mode ion‐gated OECT that exhibits both high transconductance and speedy response.^[^ ^56^ ^]^ This transistor was utilized to record non‐invasive EMG signals of muscle compound action potentials, which were triggered by the flexion of the biceps muscle (Figure 13c). The recordings were obtained with a high signal‐to‐noise ratio of 52.84 dB, indicating the high sensitivity and accuracy of the OECT in detecting muscular electrical activity. EOG signals are widely used in clinical and research settings to study eye movements and diagnose disorders of the oculomotor system, such as strabismus and nystagmus. Yao et al. successfully demonstrated a hybrid organic/inorganic complementary inverter that exhibits a high voltage gain of more than 110.^[^ ^171^ ^]^ This inverter was further utilized for the real‐time monitoring of EOG signals (Figure 13d). The high voltage gain of the inverter enabled the effective amplification of EOG signals, which can amplify the small voltage fluctuations of ≈1.5 to 30 mV, showing satisfactory performance in tracking eye movement.

EEG and ECoG are commonly used techniques for measuring brain electrical activity. EEG is non‐invasive and real‐time, aiding in the diagnosis and treatment of neurological disorders. ECoG offers higher spatial resolution and detailed information about specific brain regions. Micrometer‐scale ion‐gated OECTs can be easily attached to the skin of the head to track EEG signals.^[^ ^172^ ^]^ These transistors were able to acquire high‐quality neurophysiological signals in the alpha, beta, and gamma frequency bands, and could demonstrate the reactivity of the posterior dominant rhythm to eye closure (Figure 13e).^[^ ^172^ ^]^ In vivo detection of ECoG signals requires electrodes that are flexible and biocompatible. Khodagholy et al. were the first to apply OECT arrays for in vivo ECoG monitoring by fabricating devices on a 2 µm thick parylene film substrate (Figure 13f).^[^ ^173^ ^]^ The ultrathin OECT devices were patched onto the somatosensory cortex of rats for ECoG detection and demonstrated a high signal‐to‐noise ratio for epilepsy recordings.

## 4 Wearable and Implantable Biomarker Detections

To accelerate the translation of OECT‐based biomarker sensors into practical applications and enable point‐of‐care detection, there has been a significant focus on advancing wearable and portable sensing devices. These studies aim to integrate and miniaturize OECT devices, allowing for convenient and flexible usage by attaching them to the skin or incorporating them into fabrics specifically designed for wearable applications. Wearable devices are commonly developed to offer convenient daily health monitoring, while implantable devices are predominantly used for precise diagnostics and treatment assistance. Implantable devices face challenges related to ultra‐flexibility, biocompatibility, and degradability. Currently, implanted OECTs are primarily utilized for detecting electrical signals in the heart and cortical brain activity, as well as studying neurotransmitter transmission in the brain.^[^ ^38^, ^72^, ^73^, ^74^ ^]^

### 4.1 Wearable Biomarker Detections

Biomarker detection utilizing wearable devices primarily focuses on the identification of metabolites and surface electrophysiological signals.^[^ ^69^, ^83^, ^174^, ^175^, ^176^, ^177^, ^178^ ^]^ Metabolites are readily obtainable from bodily fluids such as urine and sweat, while surface electrical signals necessitate conduction through the human body to reach the electrode. Wearable devices predominantly encompass fiber‐based components that can be intricately woven into textiles, or flexible patches that can be affixed to the skin.

**Figure 14:**
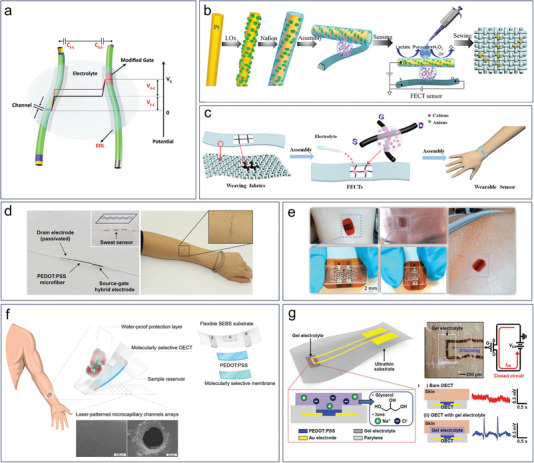
Wearable biomarker detections. a) Fiber‐based wearable OECTs for the detection of glucose. Reproduced with permission.^[^ ^43^ ^]^ Copyright 2018, Wiley‐VCH. b) Fiber‐based wearable detection of lactate. Reproduced with permission.^[^ ^117^ ^]^ Copyright 2020, Springer Nature. c) Fiber‐based wearable detection of lactate dopamine. Reproduced with permission.^[^ ^132^ ^]^ Copyright 2019, American Chemical Society. d) Single‐strand fiber‐type OECT for wearable detection. Reproduced with permission.^[^ ^181^ ^]^ Copyright 2018, Springer Nature. e) Microfluidics integrated with OECTs for efficient sweat collection. Reproduced with permission.^[^ ^138^ ^]^ Copyright 2022, American Chemical Society. f) A wearable patch combined with molecular imprinting technique for the detection of cortisol in sweat. Reproduced with permission.^[^ ^139^ ^]^ Copyright 2018, AAAS. g) Long time ECG monitoring realized by ultra‐thin OECTs with non‐volatile glycerol gel electrolyte. Reproduced with permission.^[^ ^76^ ^]^ Copyright 2019, Wiley‐VCH.

Fiber‐based OECTs often utilize smooth polymer materials, such as nylon thread, as substrates. These substrates are coated with layers of metal or organic semiconductors. A fabrication method involves using two intersecting fibers: one fiber forms the source, drain electrodes, and channel, while the other fiber acts as the gate electrode. For example, a channel can be prepared by coating a nylon thread with a spaced layer of Cr/Au and then coating it with the organic semiconductor PEDOT:PSS (**Figure** **14**a).^[^ ^43^ ^]^ A glucose oxidase‐modified Pt layer can be coated onto the other nylon thread. By connecting the two fibers with an electrolyte, glucose detection can be achieved. These two fibers can be woven into fabric or incorporated into diapers to detect glucose in urine. Similar methods can also be applied for detecting lactate in sweat (Figure 14b) and dopamine in human injection samples (Figure 14c).^[^ ^117^, ^132^ ^]^ One alternative method is to use single fibers to prepare organic electrochemical diodes by utilizing the asymmetry in the exposed metal area at both ends.^[^ ^179^, ^180^ ^]^ Kim et al. demonstrate a single‐strand fiber‐type OECT with source‐gate hybrid electrode for the detection of ion concentration in human sweat (Figure 14d).^[^ ^181^ ^]^ The PEDOT:PSS microfibers were prepared by the wet‐spinning process, whose two terminal were connected with silver wires as source drain electrodes. To form a source‐gate hybrid structure, the electrode was encapsulated with polymethyl methacrylate, leaving a section of exposed silver wire as the gate electrode. This simple structure device can respond to various concentrations of NaCl solutions. However, due to the inability to fix the device size, the uniformity of device performance may be compromised.

Devices used for detecting sweat metabolites on the skin often incorporate a sweat collection system, where sweat not only serves as the source of metabolites but also acts as the electrolyte to connect the gate electrode and the channel. In order to acquire an ample volume of sweat for the purpose of evaluating analyte concentration, microfluidics have been integrated into the system. This integration enables the efficient collection of small quantities of sweat (Figure 14e).^[^ ^138^ ^]^ Parlak et al. employed molecular imprinting technology to facilitate the detection of cortisol in sweat.^[^ ^139^ ^]^ To collect sweat from the skin, they fabricated a passive capillary‐driven fluid control system by utilizing laser patterning techniques to create 20‐µm‐wide channels on tapes (Figure 14f). Given the challenge of inducing sweat in comfortable or cold environments, the detection of sweat typically necessitates various methods to promote sweating, such as exercise,^[^ ^182^ ^]^ mild thermal stimulation,^[^ ^183^, ^184^ ^]^ or electrical stimulation.^[^ ^185^ ^]^ These techniques help facilitate the generation of sweat for effective detection purposes.

Wearable electrophysiological sensors require a flexible substrate to ensure comfort and conformability on the skin, as well as a conductive gel with optimal electrolytic properties for attaching the gate and channel to the skin. However, liquid electrolytes face challenges in adhering to the skin, while the stability of ionic hydrogels is affected by water evaporation. To overcome these challenges, Lee et al. demonstrated the use of a non‐volatile glycerol gel electrolyte containing ions as the electrolyte for ECG monitoring (Figure 14g).^[^ ^76^ ^]^ This glycerol gel electrolyte can operate over a long period, even under dry conditions. The thickness of the electrolyte can be reduced to improve the mechanical durability of the system without affecting the electrical performance of the devices.

The challenge pertaining to wearable devices lies in achieving consistent and elevated electrical performance on flexible substrates or fibers, all the while upholding a high level of sensitivity and specificity for the detection of biological signals in bodily fluids and on‐body electrophysiological signals. Several factors come into play, beginning with the careful selection of substrate materials that possess the necessary flexibility to ensure comfortable skin contact and stretchability to prevent device damage during bodily movement. Moreover, the device should exhibit high sensitivity and specificity to precisely detect minute quantities of analytes in bodily fluids while mitigating interference from other substances. Additionally, when detecting bodily fluids like tears and saliva, which are not readily accessible on the skin surface, considerations of biocompatibility and wearability become crucial. Consequently, many wearable devices are still in the experimental stage, and the development of wearable devices for tears and saliva detection remains largely conceptual at this point.

### 4.2 Implantable Biomarker Detections

**Figure 15:**
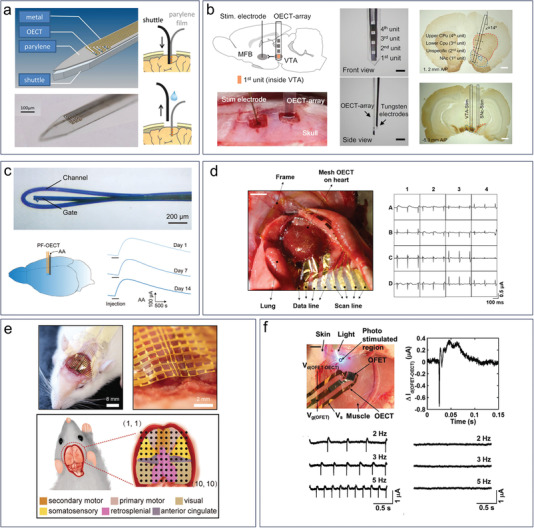
Implantable biomarker detections. a) The insertion of OECT probes into the rat brain to record neural activity. Reproduced with permission.^[^ ^72^ ^]^ Copyright 2015, Wiley‐VCH. b) Real‐time mapping of evoked neurotransmitter release in the rat brain using an OECT array. Reproduced with permission.^[^ ^38^ ^]^ Copyright 2020, eLife Sciences Publications, Ltd. c) An all‐polymer fiber‐based OECT implanted into the rat brain for ascorbic acid monitoring. Reproduced with permission.^[^ ^186^ ^]^ d) ECG signals recording on the surface of the rat heart. Reproduced with permission.^[^ ^73^ ^]^ Copyright 2018, AAAS. e) ECoG signal recording in the rat cerebral cortex. Reproduced with permission.^[^ ^74^ ^]^ Copyright 2023, Wiley‐VCH. f) Muscle action potentials recorded by flexible OECTs. Reproduced with permission.^[^ ^187^ ^]^ Copyright 2016, Wiley‐VCH.

Implantable devices are primarily used for detecting neurotransmitters in the brain and for electrocorticography (ECOG) monitoring. Neurotransmitters such as dopamine are predominantly synthesized, released within the brain, resulting in their concentration being much higher in the brain than in body fluids. Furthermore, the concentration of neurotransmitters within the brain is more stable and better reflects the actual neurotransmitter activity occurring in the brain. Williamson et al. were the first to demonstrate the insertion of OECT probes into the rat brain to record neural activity.^[^ ^72^ ^]^ The OECTs were fabricated on microprobes with dimensions of 5.3 mm in length and 200 µm in width which was composed of a SU‐8 shuttle and a 4 µm thick layer of parylene film (**Figure** **15**a). After the microelectrode is inserted into the rat brain, the SU‐8 shuttle is detached and removed. The ultra‐thin parylene film considerably reduces probe invasiveness. The OECTs were used as a current source for electrical stimulation of a specific local population of neurons, eliciting a response in one local region of the CA1. Real‐time mapping of evoked neurotransmitter release in the rat brain was successfully recorded using an OECT array.^[^ ^38^ ^]^ As illustrated in Figure 15b, a tungsten electrode was inserted into the medial forebrain bundle to evoke somatodendritic release of dopamine. An OECT array was then implanted into the ventral tegmental area, a key region involved in dopaminergic signaling, enabling mapping of evoked dopamine release in multiple striatal brain regions under different physiological conditions. Recently, an all‐polymer fiber OECT was implanted into the rat brain, achieving a stable 14‐day ascorbic acid monitoring (Figure 15c).^[^ ^186^ ^]^

In addition to detecting the release of neurotransmitters within the rat brain, OECT can also detect in vivo electrophysiological signals, including ECG signals on the surface of the rat heart (Figure 15d), ECoG signals in the rat cerebral cortex (Figure 15e), as well as muscle action potentials(Figure 15f).^[^ ^73^, ^74^, ^187^ ^]^ In order to ensure a good fit of the electrode on the tissue and reduce damage, Lee et al. fabricated an ultrathin, stretchable grid‐patterned active OECT matrix with thickness of only 2.6 µm and mapping the ECG signals of beating rat heart.^[^ ^73^ ^]^ Through coating a thin layer of poly(3‐methoxypropyl acrylate), the devices can maintain antithrombogenicity and excellent ionic conductivity. Wu et al. introduced implantable OECT arrays that can be fully biodegraded.^[^ ^74^ ^]^ Arrays of 100 channels were pattered on poly(lactic‐co‐glycolic acid) substrate, which can be degraded gradually after 2 days monitoring. This ultrathin, lightweight, and soft OECT network can map neural signals of rat cerebral cortex with high resolution. Biodegradable and biocompatible polymers exhibit favorable characteristics in terms of both human health and environmental impact.^[^ ^188^ ^]^ Biodegradable implantable devices eliminate the need for device removal or replacement from body, thereby preventing secondary injury. Wildly used polylactic acid, along with other materials like diacetate cellulose and paper, have been applied in OECT sensing as biodegradable substrates.^[^ ^40^, ^189^, ^190^, ^191^ ^]^

Compared to in vitro detection, in vivo detection provides more comprehensive information on both chemical and electrical signals. The concentration of metabolites and hormones is higher in vivo than on the body surface, and electrical signals in vivo are stronger because they do not need to be transmitted to the body surface. However, implanted devices encounter additional challenges, including the potential interference from multiple analytes and the inherent risk of body rejection. Implantable electronic devices intended for in vivo applications must be designed to minimize tissue or organ damage, refrain from impeding the activity of nerve cells, prevent inflammation, and mitigate the risk of rejection by the body's immune system. OECT fabrication offers exceptional flexibility and has demonstrated the ability to produce ultra‐thin flexible films, microelectrodes, and thin fibers with outstanding biocompatibility. The future prospects of OECTs are highly promising, particularly in the field of brain‐machine interfaces.

### 4.3 Integrated OECT Biosensor Platform

To enable practical applications, the integration of a comprehensive detection system is essential for wearable, portable, implantable, and point‐of‐care testing. The compact detection meter can be conveniently affixed to the skin, carried in a pocket, or seamlessly woven into clothing. Integrated wireless control and transmission systems typically comprise fundamental components such as a digital‐analog converter (DAC), analog‐digital converter (ADC), transimpedance amplifier (TIA), microcontroller unit (MCU), wireless communication unit (Bluetooth), OECT sensors, and mobile application software.

**Figure 16:**
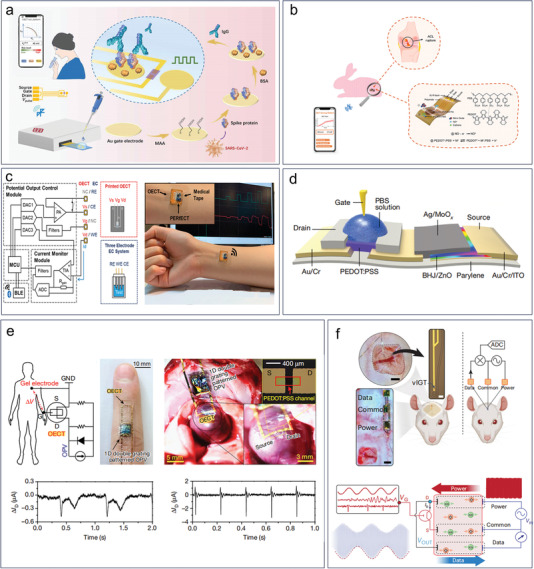
Integrated OECT biosensor platform. a) OECT‐based portable detection of SARS‐CoV‐2 IgG. Reproduced with permission.^[^ ^90^ ^]^ Copyright 2021, AAAS. b) A real‐time wireless detection of nitric oxide (NO) within the articular cavity of rabbits. Reproduced with permission.^[^ ^162^ ^]^ Copyright 2022 National Academy of Science. c) An integrated system with a size of a coin. Reproduced with permission.^[^ ^192^ ^]^ Copyright 2022, American Chemical Society. d) An OECT driven by organic photovoltaic cells. e) Self‐powered OECT for cardiac signal recording. d,e) Reproduced with permission.^[^ ^193^ ^]^ Copyright 2018, Springer Nature. f) Ion‐gated OECT powered by wireless fast alternating current for the detection of neurophysiological activity. Reproduced with permission.^[^ ^195^ ^]^ Copyright 2023, Springer Nature.

In 2021, a portable meter based on OECT was fabricated for the detection of SARS‐CoV‐2 immunoglobulin G (IgG), a crucial protein for clinical health assessment and vaccine development (**Figure** **16**a).^[^ ^90^ ^]^ By simply dropping saliva on the gate area of the OECT, the IgG level can be read on a phone within 5 min. This palm‐sized, fast, sensitive, and portable IgG meter holds great potential for combating large‐scale communicable diseases. In order to examine wound infection subsequent to ligament injury, a real‐time wireless detection system for nitric oxide (NO) was showcased within the articular cavity of rabbits (Figure 16b).^[^ ^162^ ^]^ NO‐sensitive OECT sensors were surgically implanted into the joint cavity of the rabbits and linked to a wireless module affixed to the thigh of each rabbit. This setup enabled continuous monitoring and wirelessly transmitted data for real‐time analysis of NO levels in the articular cavity. The device exhibited a low detection limit of 3 nM and high sensitivity of 94 mV dec^−1^, enabling continuous measurement of NO in chondrocytes for up to 10 hours. Selective detection was ensured by a selective membrane (poly‐5‐amino‐1‐naphthol), which demonstrated excellent selectivity for NO. As shown in Figure 16c, an integrated system was successfully fabricated that is capable of being miniaturized to the size of a coin and attached to the skin on the arm.^[^ ^192^ ^]^

The ability of OECTs to function at low voltage presents a notable advantage, as it enables the utilization of small batteries. In the past, portable or miniaturized integrated systems heavily relied on compact commercial batteries for power. However, recent advancements in research have made remarkable strides in the development of self‐powered integrated systems that eliminate the need for external power sources or cumbersome connecting wires.^[^ ^193^ ^]^ Park et al. were the first to demonstrate the integration of ultra‐flexible functional OECT devices with organic photovoltaic cells as a power source.^[^ ^193^ ^]^ The double‐grating‐patterned organophotovoltaics and OECT device were fabricated on a 1 µm thick ultra‐flexible parylene substrate (Figure 16d). The organophotovoltaics have a high power‐to‐weight ratio of 11.46 watts per gram, which is sufficient to power OECTs and enable high signal‐to‐noise ratio cardiac signal recording (Figure 16e). OECT sensors self‐powered by the organic solar cells are also used in detection of the glucose and calcium ions in tear fluids and photoplethysmogram signals.^[^ ^169^, ^194^ ^]^ Enzymatic fuel cells have the capability to transform the energy derived from metabolic processes into electrical energy. Notably, the presence of glucose in bodily fluids can generate sufficient power to operate an OECT. The detection of glucose in bodily fluids using n‐type OECTs was successfully achieved by harnessing the potential of biofuel.^[^ ^125^ ^]^ Cea and colleagues recently presented a vertical internal ion‐gated OECT, which is powered by wireless fast alternating current (Figure 16f).^[^ ^195^ ^]^ The device demonstrates the remarkable capability to acquire neurophysiological activity from the somatosensory cortex of rats in real‐time within an in vivo setting. These self‐powered electronics represent a significant advancement toward the development of next‐generation biomedical devices.

## 5 Conclusion

The OECT‐based biomarker detection platform encompasses a wide range of biomarkers, including genes, proteins, metabolites, and biophysical biomarkers, demonstrating its versatility for detecting various types of biomarkers. The high transconductance of OECTs enables on‐site amplification of electrical signals, leading to high sensitivity even with simple device construction and portable equipment. For the detection of metabolite biomarkers, OECT sensors exhibit advantages in their high sensitivity to charge transfer during redox processes. This results in a high detection sensitivity and low detection limit for small‐molecule metabolites. In the detection of electrophysiological signals, OECTs demonstrate advantages in on‐site amplification of electrical signals, achieving high signal‐to‐noise ratio and spatiotemporal resolution. Benefiting from the inherent flexibility and biocompatibility, OECT‐based biomarker sensors have been deployed into emerging wearable and implantable applications. Because of the simple device architecture and design diversity, OECTs have been integrated with other components to develop biomarker sensing platforms for practical implementations.

However, several challenges still need to be addressed in the development of practical applications of OECT‐based devices for biomarker detection. These challenges include enhancing long‐term stability, improving detection sensitivity of small molecules, advancing self‐powered detection, optimizing multiple detection capabilities, conducting clinical testing, and achieving integrated systems. First, except disposable devices, the long‐term performance stability of OECTs remains to be demonstrated, particularly for flexible, wearable, and implantable biosensors. This instability can arise from both the instability of the channel materials and the deactivation of the modified bio‐recognizers. It can also stem from the inferior mechanical performance of flexible devices. It is helpful to use highly stable channel materials such as PEDOT:PSS, which exhibit stability across a wide pH range.^[^ ^196^ ^]^ Additionally, additives can be employed to enhance channel stability.^[^ ^33^, ^197^ ^]^ Opting for electrode materials with high resistance to oxidation and considering device encapsulation can be advantageous in ensuring long‐term stability.^[^ ^198^ ^]^ Non‐wearable biosensing utilizing non‐flexible substrates is better suited for maintaining long‐term stability. In the case of biosensors, such as for metabolite detection, encapsulating enzymes within porous nanomaterials can effectively preserve the enzymatic activity and stability of the biological materials.^[^ ^199^ ^]^ Second, it is observe that the detection limits for small molecule metabolites, hormones, and neurotransmitters commonly fall within the micromolar and nanomolar range. However, the concentrations of small molecule biomarkers in blood and body fluids are frequently picomolar or even lower.^[^ ^200^, ^201^ ^]^ Furthermore, there is still a lack of enough research on self‐powered devices. The ability of wearable or portable self‐powered devices that can operate stably for extended periods requires further investigation. Due to the ease of implementation compared to bioenergy and friction‐based power generation, there is an anticipation for more applications that combine solar energy with OECT biosensors. In addition, although it is possible to detect multiple channels simultaneously in theory, there are still very few experimental studies that have demonstrated this capability in practice. Except fabricating multiple array devices on a single substrate, it is essential to have equipment capable of simultaneously detecting and recording data in order to achieve rapid multi‐channel detection. Moreover, most of the detections are currently limited to the laboratory stage and have not progressed to clinical trials. Nevertheless, the abundant clinical testing results on detecting SARS‐CoV‐2 have propelled this field forward. Lastly, it is worth noting that most flexible and wearable detection reported to date only have flexible OECT components, however, they still rely on large testing equipment. Therefore, a small portable or wearable integrated test system is indispensable for practical applications.

## Conflict of Interest

The authors declare no conflict of interest.
